# Exploring corrosion behavior, antimicrobial evaluation, molecular docking and DFT calculation of thiosemicarbazone ligand and its metal complexes

**DOI:** 10.1038/s41598-025-98580-1

**Published:** 2025-05-13

**Authors:** Howida S. Mandour, Lobna A. Khorshed, Amr M. Abdou, Basma Ghazal

**Affiliations:** 1 Physical Chemistry Department, National Research Centre, 33 El Bohoth St., Dokki, P.O. 12622, Giza, Egypt; 2 Microbiology and Immunology Department, National Research Centre, 33 El Bohoth St., Dokki, P.O. 12622, Giza, Egypt; 3 Department of Organometallic and Organometalloid Chemistry Division, National Research Centre, Giza, Egypt

**Keywords:** Thiosemicarbazone, Corrosion inhibitor, Cyclic voltammatery, Quasi-reversible, Antibacterial, DFT and docking study, Biochemistry, Chemistry

## Abstract

In the current study, the execution of thiosemicarbazone ligand (HL) as a novel corrosion inhibitor for copper metal in 1 M HCl solution was evaluated through the electrochemical measurements which includes (open circuit potential (OCP) potentiodynamic polarization (PDP) and electrochemical impedance spectroscopy (EIS). The results confirmed that the ligand (HL) acted as a good corrosion inhibitor for copper metal in 1 M HCl solution; as it displayed high percentage of inhibition efficiency about 94.66% and 92.93% after PDP and EIS methods respectively; at its optimum concentration (1 × 10^–7^ M). The morphology and surface constituents of the sample were examined before and after addition of the ligand (HL) by using the analysis (scanning electron microscope and an energy dispersive X-ray spectroscopy) which clarified the passivation effect of the ligand (HL) after formation of a protective layer of its adsorbed molecules on the surface of the copper sample. In addition, the metal complexes Ni (II), Co (II) and Cd (II) derived from thiosemicarbazone ligand (HL) were used in this study to shed light on some of their electrochemical properties. But based on their nature as they are insoluble in aqueous media the cyclic voltammetry method was used in this section. The results deducted from cyclic voltammetry technique showed that, the oxidation–reduction process of the ligand (HL) and its metal complexes Ni (II), Co (II) and Cd (II) under quasi-reversible system and the reaction occurred on the metal surface under diffusion control. In vitro, the antibacterial activity testing against *S. aureus, S. pneumonia, E. coli and S. Typhimurium* were performed for the ligand (HL) and its metal complexes Ni (II), Co (II) and Cd (II). The result showed that Co (II) and Cd (II), complexes exhibited the best antibacterial activity against *S. pneumonia, S. Typhimurium and E. coli* while, all the compounds did not show any antibacterial activity against *S. aureus*. To obtain a good relation that supports and explains the interactions between the molecules of the studied compounds and the metal surface and with the antibacterial activity; the theoretical study in detail was applied using density functional theory (DFT) and molecular docking. The parameters such as, energy level (ΔE), the highest HOMO (E_H_), and the lowest occupied LUMO (E_L_), molecular orbital and the binding energy are deducted and discussed. The main target investigated of this study is that the thiosemicarbazone ligand (HL) can be used as a new corrosion inhibitor for the metals and their alloys against the aggressive media. Also, from cyclic voltammetry technique which had been used for testing the metal complexes Ni (II), Co (II) and Cd (II) derived from the ligand (HL); all the details about the redox reactions of these compounds had been obtained. The importance of knowing oxidation and reduction reactions is due to their consideration as the main source of energy for the most biological process, energy productions, photosynthesis to immune responses and the synthesis and breakdown of biomolecules. Therefore, redox reactions are very important in our life.

## Introduction

Since ancient times, the use of metals and their alloys in various aspects of life are considered one of the main pillars on which mankind relied especially, in various fields of industry. In the present study, copper metal was used due to its distinctive properties such as; flexibility, elasticity, hardness, color, low cost, electrical conductivity and resistance to corrosion^1,2^. However, the exposure of these metals to the corrosion process which in turns leads to the transformation of these metals into more stable state and therefore they were consumed or dissolute when exposed to the aggressive environments resulted in a serious problem^3,4^. So, many experiments have been carried out to find the best techniques for controlling or preventing the bad effects of the corrosion process which appeared onto the uncover metals risked to rough environments^5,6^.

Currently, the method of adding inhibitor is one of the most inexpensive, most effective and common methods to reduce the rate of corrosion and they are often added in small quantities^7,8^. The effectiveness of these compounds to act as the corrosion inhibitors depending on the presence of nitrogen, phosphorus, oxygen and sulfur atoms in their structure resulted in increase the electron density of these compounds^9^ The performance and interaction of these inhibitors comes through the adsorption of their molecules onto the metal surface depending on their charge and the surrounding medium which may be (basic, acidic or neutral). As a result, the active sites of the metal surface had been blocked; therefore, the corrosion process was hindered. Also, based on the physicochemical properties of these inhibitors such as; electron density of the electrons, the molecular and electronic structure of the molecule^10,11^. Among of these compounds that were widely used in different fields was the thiosemicarbazones. For recent time, thiosemicarbazones has been used as a perfect organic corrosion inhibitor^11^. Also, thiosemicarbazones has been used widely in medicinal, pharmacological and biological applications which included cytotoxic, antitumor and antibacterial^12,13^. In addition, in laboratory it was successfully used as analytical reagent and chelating agent^14^. And, according to previous studies many uses and importance of the thiosemicarbazones are due to its chemical structure^12,15–17^. Therefore, in the present work the use of thiosemicarbazones derivative (ligand HL) as a corrosion inhibitor had been highlighted dependent on the chemical structure of the thiosemicarbazones and its various applications. So, the synthesized thiosemicarbazone ligand (HL) was examined to be used as a new corrosion inhibitor for copper metal in the corrosive medium (1.0 M HCl). As well as studying some of the electrochemical properties of the ligand (HL) and its Ni (II), Co (II) and Cd (II) complexes, by using cyclic voltammetry technique. In vitro*,* the test of the antibacterial activity of the ligand (HL) and its Ni (II), Co (II) and Cd (II) complexes were carried out to discover the validity of them as antibacterial compounds. To get more details and a new idea about the previous studies of thiosemicarbazone ligand (HL) and its Ni (II), Co (II) and Cd (II) complexes; a theoretical study including density functional theory (DFT), and molecular docking were evaluated and discussed.

## Experimental work

### Materials and methods

All the materials used for the preparation of the thiosemicarbazone ligand (HL)[(E)-N/-(1-(4-aminophenyl) ethylidene) morpholine-4-carbothiohydrazide] and its metal complexes were of analytical grade (BDH, Merck and Sigma Aldrich) by means with high percentage of purity. The metal complexes which had been studied in the current work include: -

[(E)-N/-(1-(4-aminophenyl) ethylidene) morpholine-4-carbothiohydrazide-Ni(II)], [(E)-N/-(1-(4-aminophenyl) ethylidene) morpholine-4-carbothiohydrazide-Co(II)], [(E)-N/-(1-(4-aminophenyl) ethylidene) morpholine-4-carbothiohydrazide Cd(II)].

For instance, p-aminoacetophenone (4-Aminoactophenone 99%), ethanol (95.5%), anhydrous CaCl_2_ (98%), the metal salts (CoCl_2_, NiCl_2_ and CdCl_2_ (99%)) and H_2_SO_4_ (99.9%). The compound 4-morpholinethiosemicarbazide (hydrazine carbothioamide) was prepared by the authors according to the literature^18–22^.

Thiosemicarbazone ligand (HL) and its metal Ni(II), Co(II) and Cd(II) complexes were prepared and their structural properties were studied and interpreted in several methods according to the published literature^22^. Open circuit potential (OCP), electrochemical impedance spectroscopy (EIS), Potentiodynamic polarization (PDP) and cyclic voltammetry are the electrochemical methods used in this study by using Autolab potentiostat/galvanostat PGSTAT302N. These methods were carried out to test the efficiency of the ligand (HL) as a new corrosion inhibitor for copper metal in the aggressive medium (1 molar HCl). And, to deduce some electrochemical behavior of the ligand (HL) and its Ni (II), Co (II) and Cd (II) complexes.

Disc diffusion method was used to evaluate the antibacterial activity of the ligand (HL) and its Ni (II), Co (II) and Cd (II) complexes against four pathogenic strains of both gram- positive and gram- negative bacteria. Computational methods are applied which gave important evidence about the reactivity center and biological efficiency of the thiosemicarbazones ligand (HL) and its Ni (II), Co (II) and Cd (II) complexes.

Preparation of the thiosemicarbazone ligand (HL) and its metal complexes Ni (II), Co (II) and Cd (II).

Depending on the experimental data that had been presented in the published literature^22^; the thiosemicarbazone ligand (HL) was synthesized as following: (1) In hot ethanol 0.1 mol of *p-*aminoacetophenone (13.52 g) was added of to 0.1 mol of 4-morpholinethio semicarbazide (16.12 g). (2) After one hour of begging the reaction; few drops of concentrated H_2_SO_4_ acid were added to catalyze the reaction mixture. (3) The product (yellow precipitate) was filtered and washed many times with ethanol and then dried in a vacuum desiccator contains anhydrous CaCl_2_.

The metal complexes obtained from the reaction of the ligand (HL) with appropriate metal salt had been prepared in two molar ratios (1L:1 M) and (2L:1 M). The metal complexes Ni (II), Co (II) and Cd (II) which had been studied in the current work were prepared in two molar ratios (1L:1 M), for Ni (II), Co (II) complexes and (2L:1 M), for Cd (II) complex. Ni (II), Co (II) and Cd (II) complexes have been synthesized according to the following steps: (1) Dropwise addition of 0.01 mol of ethanolic solution (35 ml) of metal chloride MCl_2_.nH_2_O: M (Ni (II), Co (II) & Cd (II)) to 0.01 mol and 0.02 mol of hot ethanol solution from the ligand (HL), to prepare Ni (II) and Co (II) complexes in molar ratio (1L:1 M), and to prepare Cd (II) complex in molar ratio (2L:1 M), respectively. (2) For about four hours at 80 °C the reaction mixture was refluxed. (3) The products were filtered and washed with ethanol several times and then dried in a vacuum desiccator containing anhydrous CaCl_2_.

In most previous studies, the changing of molar ratios had been affected on stoichiometry of the complexes. For example, building on the published paper referenced with number twenty-two^22^; the metal complex Zn (II), was prepared in the two molar ratios; (1L:1 M) and (2L:1 M). So, it can be observed from Table 1 the effect of changing the molar ratios for Zn (II) complex as instance^22^.

**Table 1:** Comparison between the formula weight, the percentage of elemental analyses, melting points and molar conductance for Zn(II) complex derived from the ligand (HL) in two molar ratios.

No	Compound	F.W	Elemental analyses found/(calc)%						M.P. (°C)	*Λ* (Ω^−1^ cm^2^ mol^−1^)
			C	H	N	S	Cl/Br	M		
1	[Zn(HL)Cl_2_(H_2_O)_2_]. H2O C_13_H_24_N_4_SO_4_ZnCl_2_	468.84 Yellow	33.77 (33.30)	4.99 (5.16)	12.09 (11.95)	6.81 (6.84)	15.73 (15.14)	13.58 (13.95)	187	6
2	[Zn(HL)_2_Cl_2_].1.5H_2_O C_26_H_39_N_8_S_2_O_3.5_ZnCl_2_	720.19 Yellow	43.81 (43.36)	5.40 (5.46	15.13 (15.56)	8.25 (8.90)	9.13 (9.86)	9.21 (9.08)	174	7

1- (1L:1 M) and 2- (2L:1 M).

**Fig. 1:**
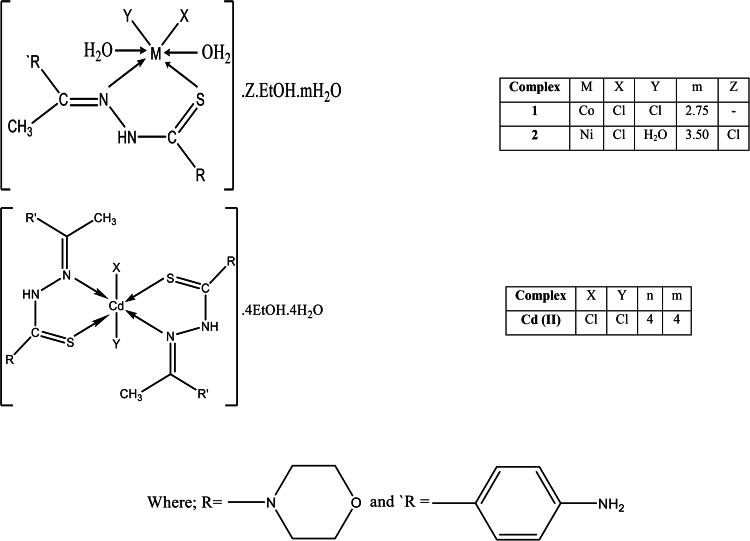
The proposed chemical structure for metal complexes Ni (II), Co (II) and Cd (II).

In addition, the ^1^HNMR is similar to the published data^22^. Table S2, it can be seen that, the metal complex Zn(II), which was prepared in molar ratio (1L:1 M), was separated in *E* isomer only while the Zn(II) complex in molar ratio (2L:1 M), had existed in *Z/E* isomer complex and both were confirmed to have octahedral structural. The submitted structures of the ligand (HL) and its (Ni (II), Co (II) and Cd complexes had been presented in Fig. 1.

All the characterizations of the ligand (HL), and its metal complexes Ni(II), Co(II) and Cd(II) have been presented as supporting information in the supplementary file. For example, the analytical and spectral studies (IR, ^1^H NMR, mass), which proved that the ligand (HL) chelated in a neutral bidentate pattern through the azomethine nitrogen and thione sulfur atoms; had been shown in Figs. (S1, S2) and Tables (S1, S2, and S3). In addition, the electronic spectra and magnetic moment emphasized the stereochemistry of the metal complexes had been shown in Table (S4). As well, the stability of the prepared thiosemicarbazone ligand (HL) and its metal complexes which was deduced from their thermal analysis (TG, DTG), had been displayed in Fig. (S3) and Table (S5). The results obtained from the TG curves demonstrated that, the studied metal complexes in the present work (Ni (II), Co (II) and Cd (II)), had been shown higher thermal stability than the free ligand (HL).

### Corrosion measurement

The testing of the corrosion process was done onto pure copper sheet 1.0 cm^2^. In a conventional three-electrode cell contains silver/silver chloride as a reference electrode, platinum wire as a counter electrode and copper samples as a working electrode. The electrochemical methods include (open circuit potential, potentiodynamic polarization and electrochemical impedance spectroscopy), which had been performed by using an Autolab potentiostat/galvanostat PGSTAT302N. Firstly, the copper samples were abraded with emery papers of grades 600, 800, and 1200 after that degreased in acetone then washed thoroughly with double-distilled water and finely dried. The copper samples had been immersed in the corrosive medium (1.0 M HCl) with different concentrations of the thiosemicarbazone ligand (HL) [1 × 10^–7^, 1 × 10^–6^, 1 × 10^−5^and1 × 10^–4^ M] for 60 min; until steady state potential was reached. After that, the polarization process was carried out at scan rate of 1 mV/sec and at room temperature. It can be noticed that, in the corrosion test the ligand (HL) was only used as a corrosion inhibitor without using its Ni(II), Co(II) and Cd(II) complexes. This observation was attributed to that; these metal complexes are insoluble in the aqueous medium.

The electrochemical impedance spectroscopy (EIS) assessments were recorded after immersion of the working specimen in the tested solution for 60 min in the frequency range from 100 kHz to 0.01 Hz and the AC signal was 10 mV peak to peak. After that, the impedance data was resolved and fitted.

### Cyclic voltammetry study

Cyclic voltammetry technique was applied to deduce some of the electrochemical properties of thiosemicarbazone ligand (HL) and its metal Ni (II), Co (II) and Cd (II) complexes by using Autolabpotentiostat/galvanostat PGSTAT302N at 25 °C. A glass cell with three electrodes was used in this work; silver/silver chloride as a reference electrode, platinum wire as a counter electrode and platinum rod as a working electrode. The laboratory experiment was carried out by preparing 0.005 M solution of the ligand (HL) or its metal complexes with 0.05 M of tetra- butyl ammonium perchlorate (TBAP) in dimethyl sulphoxide (DMSO). The potential range for all the experiments of (HL), and its complexes was recorded between (− 1.5 and** + **1.5 V) versus Ag/AgCl with different scan rates (50–500 mV s^−1^) under nitrogen atmosphere. All the voltammograms were recorded under the above atmosphere at the room temperature.

### Antibacterial activity

The antibacterial activity of the prepared compounds was evaluated *in vitr*o against 4 pathogenic strains of both gram- positive and gram- negative bacteria. gram- positive strains were represented by *Staphylococcus aureus* and *Streptococcus pneumoniae* while gram- negative strains were represented by *Escherichia coli* and *Salmonella Typhimurium* using disc diffusion method on Muller-Hinton agar. The test oorganisms were maintained on agar slant at 4 °C and subculture on fresh agar plates. For disc diffusion assay, bacterial liquid cultures were initiated by placing a loop of bacteria into 10 ml of lysogenic broth (LB) media. Agar diffusion test was conducted to detect the bacterial susceptibility to the prepared compounds^23^. A volume of 100 µL of cell culture suspension matching with 0.5 McFarland of each test organism were spread onto the surface of solid agar medium (Muller Hinton agar).

The prepared compounds were adjusted to a concentration of 50 mg/mL using DMSO as solvent. Filter paper discs with a diameter of 7 mm each were impregnated with 15 µL of each of the different compounds. Ofloxacin discs (OFX-5) were used as standard antibiotics and filter paper discs impregnated with 15 μL of DMSO were also used as control for the solvent. Then the agar plates containing microorganisms soaked with paper discs (5 µg) were incubated at 37 ± 0.1 °C for 24 h to allow bacterial growth. After incubation, zones of inhibition were measured as the clear zones around each disc in mm using a ruler. The experiment was carried out in triplicates for statistical relevance and the Mean ± SE of results was calculated.

### Surface analysis

The surface morphology and composition of the copper sample was investigated after it was examined in the tested solution (1.0 M HCl) without and with the optimum concentration of the thiosemicarbazone ligand (1 × 10^−7^ M). Scanning electron microscope (SEM) with energy dispersive X-ray spectrometer (EDX) analyses were used to achieve the above purpose by (SEM/ EDS) Model FEG Quanta 250, Holland.

### Computational methods

The starting geometry of the ligand (HL) and all studied complexes (Ni (II), Co (II) and Cd(II)) were prepared via GaussView 6^24^. and optimized using Gaussian 16, Revision C.01^25^, using DFT/CAM-B3LYP method and LanL2DZ basis sets^26,27^. Frequency calculations had been performed to ensure the absence imaginary frequencies at the same level of theory. Frontier molecular orbitals had been studied in the ground state to get a conclusion about the electronic natures of the studied compounds. Electrostatic potential maps^28^ were analyzed to identify the reactivity centers of the ligand and the studied complexes.

Electronic parameters derived from DFT, such as, energy of HOMO (E_H_), energy of LUMO (E_L_), band gab (ΔE), absolute softness (S)^29^; which indicate the capability of the ligand or complex to form a covalent bonds, global hardness (η); that related to the struggle of the system to exchange electronic charge with the environment, electrophilicity index (ω); which describes how does the stabilization energy of a given system affected via gains an additional electronic charge from the surrounding, nucleophilicity index (*N*); that is the inverse of the electrophilicity, (1/ω), chemical potential (µ); that regulates the tendency of the electrophile to accept more electronic charge are all calculated as the following^30–32^

1 $$\Delta {\text{E}} = {\text{ E}}_{{{\text{LUMO}}}} - {\text{E}}_{{{\text{HOMO}}}}$$

2 $${\text{I}} = - {\text{E}}_{{{\text{HOMO}}}}$$

3 $${\text{A}} = - {\text{E}}_{{{\text{LUMO}}}}$$

4 $$\eta = \left( {E_{HOMO} - E_{LUMO} } \right)/2$$

5 $$\sigma = 1/\eta$$

6 $$\mu = - \left( {1 + A} \right)/2$$

7 $$X = \left( {I + A} \right)/2$$

where E_HOMO_ and E_LUMO_ represent the energies of HOMO and LUMO, respectively and ΔE, is the band gap.

### Molecular docking

Molecular docking was conducted for the ligand (HL) and all studied complexes to explore their biological efficiencies toward ribosyltransferase (PDB ID: 3GEY). The AutoDock 4.0 software package was employed to run molecular docking using the crystal structure downloaded from protein data bank. The binding site was explored using a discovery studio using central ligand inside the cavity of protein.

Steps of docking were followed the following procedure^33–35^:

### Ligand preparation

The optimized geometry obtained from gaussian was prepared using discovery studio after download of the protein from protein data bank. Routinely process was performed to add hydrogen and gastegier charges.

### Protein preparation

The structure of ribosyltransferase (PDB ID: 3GEY) was obtained from the protein data bank RCSB (https://www.rcsb.org/). Then, the discovery studio software was used to identify and visualize the active site with the studied protein. The structure of (3GEY.pdb) was isolated from the ligand and cleaned from all heteroatoms and water molecules to ensure that the active site is free from ligand before docking process. The hydrogen atoms and Kollmann charges were added automatically from the Auto Dock software.

### Receptor grid generation

Automatically generation of the grid via Auto Dock 4.0 had been processed by selecting the active site residues of protein (PDB ID: 3GEY).

### Docking via auto dock 4.0

The interaction mode of the ligand (HL) and all studied complexes were predicted in the active site of 3GEY through rigid docking via Auto Dock 4.0. The ligands were docked onto the active site through default setting of Lamarckian genetic algorism. After docking, the individual binding conformations of each ligand were observed, and the interaction with the protein were studied. The best and most energetically favorable conformation structure was selected based on the best fitness function detected through the algorism. Then, the resulting docked structures were quantified in terms of binding energy (Kcal/mol). The predicted binding free energy was used as criteria for ranking the compound potency.

## Results and discussion

### Open circuit potential test (OCP)

**Fig. 2:**
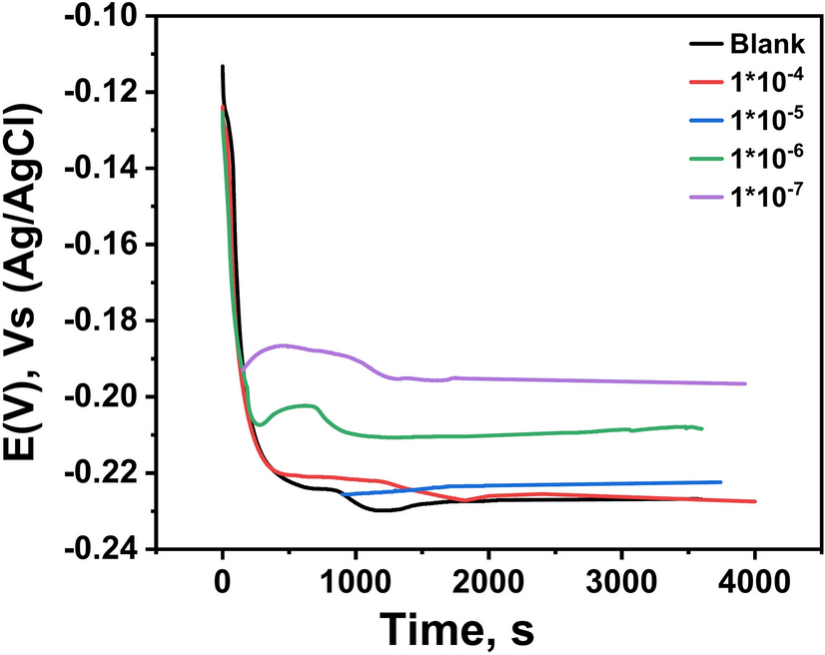
Open circuit potentials (OCP) as function of time for copper metal in 1.0 M HCl solution without and with different concentrations of the inhibitor (HL) recorded at 25 °C.

The values of the open circuit potential (OCP) of the copper samples in a solution of (1.0 M HCl) in absences and presence of the inhibitor (HL) were measured and drawn as shown in Fig. 2. Based on Fig. 2, the values of the open circuit potential (OCP) had been shifted in a positive direction from (− 0.236 to − 0.189 V) at concentrations (1 × 10^–4^, 1 × 10^–5^, 1 × 10^–6^ & 1 × 10^−7^ M) of the inhibitor (HL). This result was attributed to the adsorption of the inhibitor molecules (HL) onto the copper surface resulted in formation of a protective film^36^. Therefore, the active sites of the sample surface had been blocked because of the deposited film which led to hindering the penetration of the chloride ions to the metals surface.

### Potentiodynamic polarization studies

**Fig. 3:**
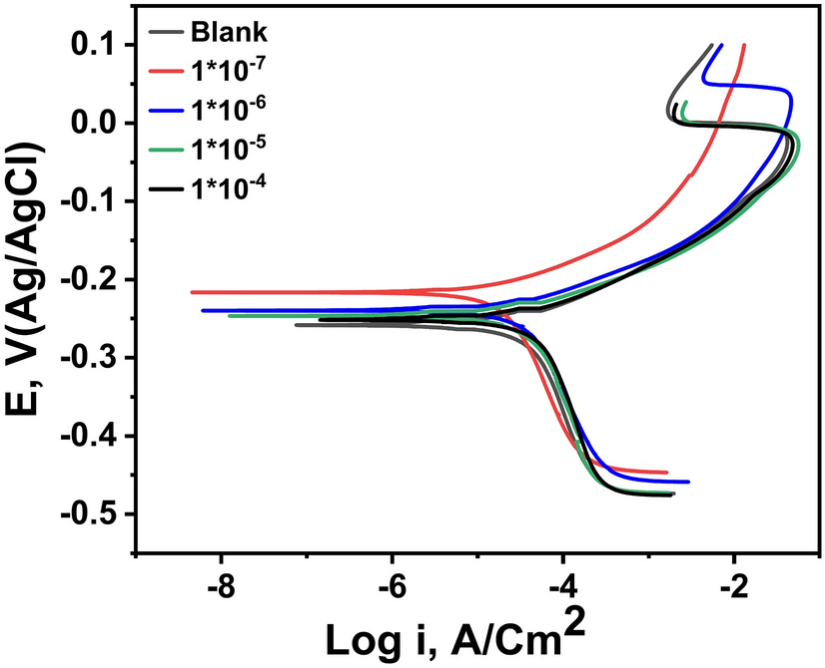
Polarization curves of the copper metal in 1.0 M HCl solution containing various concentrations of the inhibitor (HL) recorded at 25 °C.

The anodic and the cathodic Tafel curves of thiosemicarbazone ligand (HL) had been investigated after the potentiodynamic polarization method (PDP) was carried out to understand the behavior of the ligand (HL) as a corrosion inhibitor. After inspection and investigation of the results, Fig. 3, showed the effect of adding different concentrations of thiosemicarbazone ligand (HL) to 1.0 M solution of HCl as the corrosive medium onto the copper metal. It can be noted that, both cathodic and anodic branches were safely changed in another meaning the addition of the inhibitor (HL) affected on the corrosion mechanism of the electrolyte (1.0 M HCl) by retarding or inhibiting the hydrogen evolution and the metal dissolution reaction^37^.

Table 1 shows the electrochemical polarization parameters (the corrosion potential (*E*_*corr*_) the corrosion current density (*i*_*cor*r_) the cathodic and the anodic Tafel slopes (*β*_*c*_ & *β*_*a*_) and the corrosion rate (*CR*)), which had been deduced from the experimental data of Fig. 3. Depending on Fig. 3, and on the date listed in Table 1, it can be observed that, the corrosion rate of the copper sample recorded the lowest value (0.0009 mm per year) for 1.0 M solution of HCl contains 1 × 10^−7^ M concentration of the inhibitor (HL) as it was considered the optimum concentration. The percentage of the inhibition efficiency was calculated using Eq. (8) which exhibited the maximum value about (94.66%) at the ideal concentration of (HL).

8 $$IE\% = \frac{{i_{corr}^{o} - i_{corr} }}{{i_{corr}^{o} }} \times 100$$

where ${i}_{corr}^{o}$ and ${i}_{corr}$ are the corrosion current density of the copper sample in the solution 1.0 M HCl without and with different concentrations of the inhibitor (HL) respectively.

While an inhibitor concentration higher than 1 × 10^−7^ M was used, the corrosion rate reported higher value and therefore the percentage of calculated inhibition efficiency was decreased. This result was clearly attributed to the active sites of the copper surface i.e. when these active sites of the metal completely covered by the adsorbed inhibitory molecules; hence there is no chance for more inhibitory molecules to be adsorbed so any increase of the inhibitor concentration does not change the efficiency^38^. However, it was noticeable that the corrosion process still occurred; this result means that the protective layer does not change but the corrosion activity increased against the immersion time. Therefore, because it was becoming clear that the concentration factor effects on the corrosion process so the different added concentrations of the inhibitor (HL) would be arranged according to increase of the percentage of the inhibition efficiency or decreasing the values of the corrosion rate as follows: [1 × 10^–7^ > 1 × 10^–6^ > 1 × 10^–5^ > 1 × 10^–4^ M]. Also, there are another factors effected on the rate of corrosion and the inhibition efficiency such as; the mechanism of protection, types of inhibitor and their structure^39^.

It was cleared from Table 1, that the corrosion current density (*i*_*corr*_) recorded the lowest value 0.086 µA/cm^2^ at the concentration 1 × 10^–7^ M of the inhibitor (HL) beside it can be noticed the decreasing of the cathodic and anodic current values (*β*_*c*_ & *β*_*a*_) in presences of different concentrations of the inhibitor (HL) in comparing with the blank. This result indicates that the addition of the inhibitor (HL) affected the reactions of the system, which includes the hydrogen evolution reaction and metal dissolution reaction.

Through the data recorded in Table 1, and Fig. 3, it was observed that the cathodic slope offered slight displacement in comparing with the anodic slope; this result indicates that the anodic reaction was more inhibited by meaning that the addition of the inhibitor (HL) prevent in a great extent the hydrogen evolution reaction and also retard the anodic dissolution of copper metal. Therefore, the anodic current can be explained in more detail as follows; the presence of the chloride ions during the polarization process simulates the anodic current to demolition of the localized anodic oxide film and encourage formations of the copper chloride film. This result indicated that, during the anodic period the anodic current became far from the corrosion front, therefore, gave a chance for cathodic activity which led to inhibit the hydrogen evolution and then hindering the corrosion process^40^. In addition, the anodic behavior can be also interpreted as follow; the anodic period exhibits three anodic regions: (1) the region near or close to the corrosion potential *E*_corr_ called the active dissolution region as a result of the anodic dissolution of the copper metal (2) the second region called the passivity region as a result of formation the copper chloride film and (3) the third region called the dissolution of the anodic passive layer^41^.

Therefore, the reaction of the copper metal in this acidic medium 1.0 M HCl can be explained according to the following equation:

9 $$2Cu+4HCl+{O}_{2}\rightleftharpoons {Cu}^{+2}+4{Cl}^{-}+2{H}_{2}O$$

Thus, this result refers to that the inhibitor (HL) can act as mixed- type inhibitors mostly anodic type inhibitors^42,43^. It was noticeable from the values of the corrosion potential (*E*_*corr*_) in Table 1, that it does not show regular pattern towards positive or negative direction beside the shifting of their values to less than 85 mV in comparing with its value to the blank, this result confirms the use of the inhibitor (HL) as mixed-type inhibitors^44,45^.

### Electrochemical impedance spectroscopy (EIS)

**Fig. 4:**
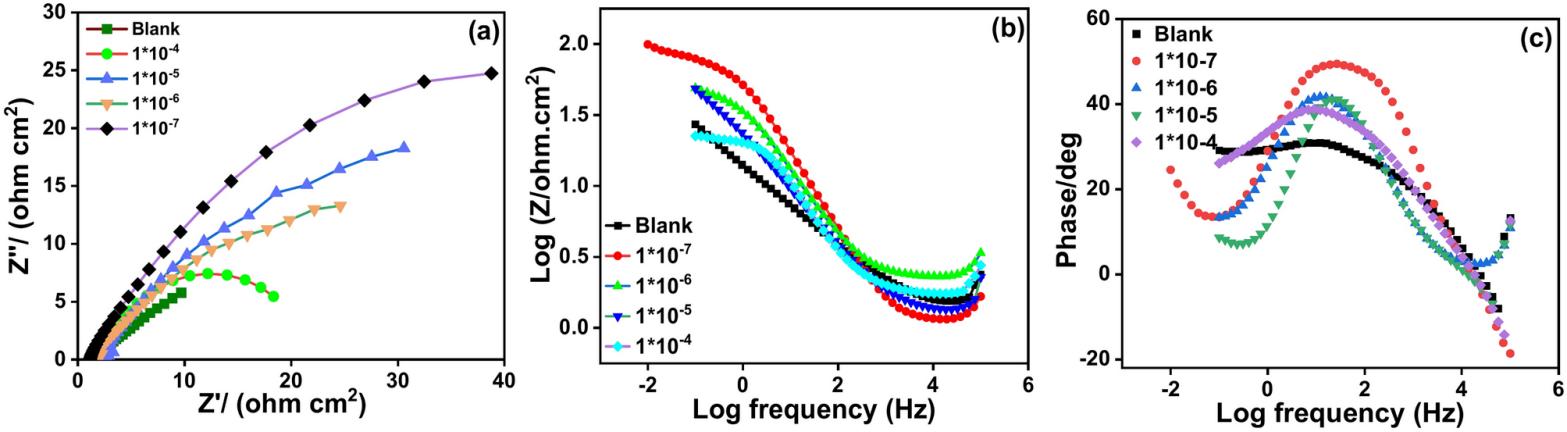
(**a**) Nyquist (**b**) bode modulus and (**c**) bode phase angle plots of the copper metal in 1.0 M HCl solution containing different concentrations of the inhibitor (HL) at 25 °C.

Corrosion mechanism, surface properties, adsorption process and supporting the data obtained by potentio-dynamic polarization method can be confirmed by powerful technique which was (EIS)^46^. In this method, the corrosion process of the copper samples in a solution of 1.0 M HCl in the presence and absences of various concentrations of the inhibitor thiosemicarbazone ligand (HL) was investigated at room temperature 25 °C. The impedance spectra Nyquist and Bode plots of the copper specimens without and with the inhibitor (HL) in acidic medium 1.0 M HCl were investigated and reported in Fig. 4. The spectrum of Fig. 4a showed one capacitive loop which reached maximum size after addition of the concentration 1 × 10^–7^ M of the inhibitor (HL) to solution1.0 M HCl.

This result indicates that the substrate impedances the corrosion process by forming a protective layer of the adsorbed inhibitor molecules on the copper surface. Also, the appearance of the curves of the Nyquist Fig. 4aby one capacitive loop proved that the charge transfer was the controlling factor in the corrosion process^47,48^.

The experimental parameters of EIS are established in Table 2. It can be concluded from Fig. 4a, and Table 2, that the capacitive loop size and the charge transfer resistance (*R*_*c*t_) had been increased according to the concentrations of the inhibitor (HL) as follows: [1 × 10^–7^ > 1 × 10^–6^ > 1 × 10^–5^ > 1 × 10^–4^ M]. As can be seen from Table 2, that the charge transfers resistance *(R*_*ct*_) reached the highest value 92.93 Ω cm^2^ at the concentration 1 × 10^−7^ M of the inhibitor (HL). And, by comparing the results deduced from PDP method and with the results obtained from EIS method; the matching between the two dates can be observed.

**Table 2:** Corrosion parameters of the copper samples after being examined in a solution of 1.0 M HCl contain various concentrations of the inhibitor (HL) at 25 °C.

	Conc., M	*β*_*a*_, mVdec^-1^	*β*_*c*_, mV dec^−1^	− *E*_*cor*r_, mV	${i}_{corr}^{o}$, μA cm^−2^	$IE\%$	*C.R*., mm/year
Blank	–	70.285	40.553	244.47	1.610	–	0.0186
Various conc. of the inhibitor (HL)	1 × 10^–4^	50.635	36.600	196.531	0.254	84.22	0.0029
	1 × 10^–5^	40.860	37.610	246.750	0.176	89.07	0.0019
	1 × 10^–6^	34.190	33.260	239.860	0.143	91.12	0.0016
	1 × 10^–7^	24.700	32.870	216.512	0.086	94.66	0.0009

This result indicates that the redox reactions occurred slowly and the charge transfer resistance more difficult so it was prevented the charge transfer at the metal/solution interface i.e. penetration of the copper surface by the chloride ions had been controlled^49–51^.

**Fig. 5:**
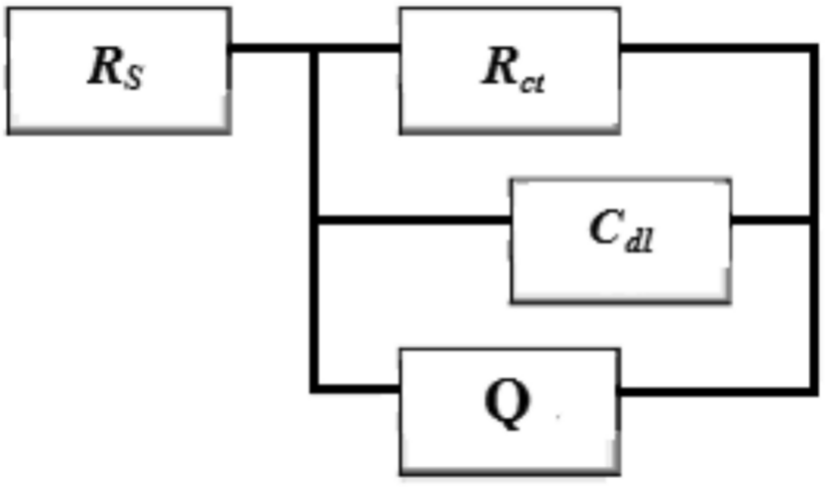
Equivalent circuit model used to fit the impedance data (EIS).

The percentage of the inhibitor efficiency was calculated according to the following equation:

10 $$IE\% = \frac{{R_{ct}^{o} - R_{ct} }}{{R_{ct}^{o} }} \times 100$$

where ${R}_{ct}^{o}$ and ${R}_{ct}$ are the charge transfer resistance with and without the inhibitor (HL) respectively. According to the EIS method, and as be expected the Nyquist plots will be given a perfect semicircle but it does not appeared in that way; in general this deviation was related to the frequency dispersion, the adsorption of chloride ions on the surface, the factors of roughness and heterogeneity of the sample surface^52–54^. Obviously, the Bode plots Fig. 4b, displaced a single phase with higher values in the region of low frequencies. This result proves that there was one-time constant which explained the reactions occurred onto the copper surface. In addition, it can be observed from Fig. 4c, that, the phase angle showed displacement approximately 80° which confirms the presences of a protective layer of the inhibitor molecules presented on the sample surface. This protective layer resulted in reducing the surface heterogeneity by blocking the most active sites and then impeded the adsorption of the chloride ions^55,56^ as well there was no possibility for the diffusion process^57^. A simple equivalent circuit modal in Fig. 5, was used to analyze the experimental data of the EIS spectra for the copper samples in 1.0 M solution of HCl without and with the inhibitor (HL) where (*R*_ct_) is the charge transfer resistance (*R*_s_) the solution resistance (*C*_dl_) the double layer capacitance and (Q) refers to the capacitance of the part that absorbed the inhibitor molecules.

The double layer capacitance *C*_dl_ was calculated according to the following equation:

11 $$f(-{Z}_{max}^{//})=\frac{1}{2\pi {C}_{dl}{R}_{ct}}$$

where the $(-{Z}_{max}^{//})$, is the maximum imaginary component of the impedance. 

In addition, it can be seen from Table 2, that the double layer capacitance was decreased in presence of the inhibitor (HL) which occurred as a result of a decrease in the dielectric constant^58^ or as a result of increase in thickness of the double layer formed on the surface of the metal. This result indicates that, the inhibitor (HL) molecules had been adsorbed at the copper/solution interface^59^.

### Cyclic voltammetry studies of the ligand (HL) and its metal Ni (II), Co (II) and Cd (II) complexes

The oxidation and reduction reactions of ligand (HL) and its Ni (II), Co (II) and Cd (II) complexes of concentration 0.005 M with 0.05 M of tetra-butyl ammonium perchlorate in dimethyl sulphoxide (DMSO) with potential range between − 1.5 and + 1.5 V with different scan rates 50–500 mV s^−1^.

#### CV study of the ligand (HL)

**Fig. 6:**
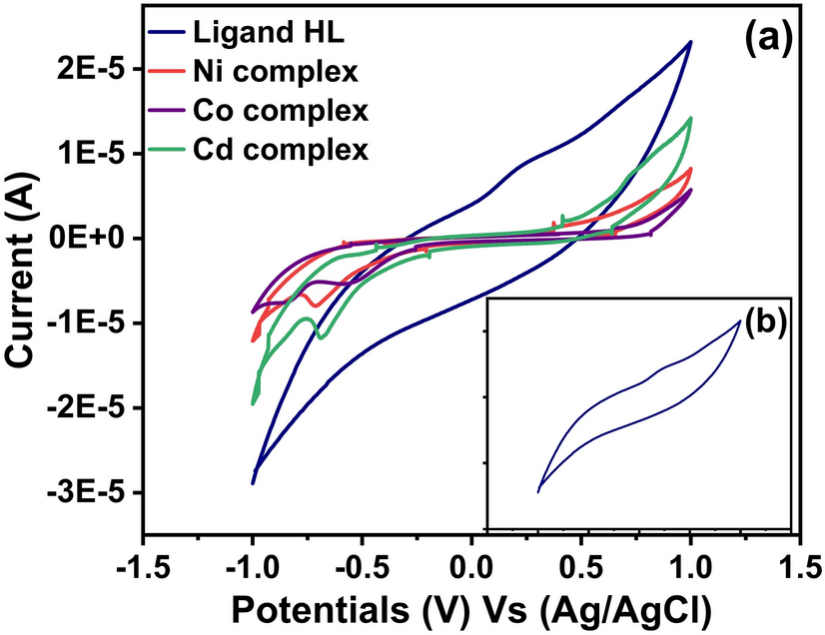
Cyclic voltammograms of 0.005 M solution of the ligand (HL) and its Ni(II), Co(II) and Cd(II) complexes with 0.05 M solution of TBAP in DMSO at scan rate 50 mV s^−1^.

Figure 6, shows the cyclic voltammograms of the ligand (HL) and its Ni (II), Co(II) and Cd (II) complexes at scanning potential ranged between − 1.5 and** + **1.5 V and scan rate 50 mV s^−1^. It can be observed that for the ligand (HL) there were two oxidation peaks E_pa1_ at + 0.041 V and E_pa2_ at + 0.231 V that attributed to the oxidation of amine group (NH & NH_2_), and the broad reduction peak E_pc_ at − 0.394 V which may be related to the reduction of azomethine or thione groups^60–64^.

#### CV study of Ni (II) complex

The cyclic voltammogram of Ni (II) complex at scan rate 50 mV s^−1^ in Fig. 6, presented one oxidation peak at + 0.471 V corresponding to the oxidation of Ni (II) to Ni (III) and one cathodic peak at − 0.707 V attributed to the reduction of Ni (III) to Ni (II)^65^.

After comparing between the position of the oxidation peak for the ligand (HL) and its Ni (II) complex it can be noted that, the value of oxidation peak increased in a positive way of the Ni (II) complex more than the ligand (HL)^63^ as well, the disappearance of azomethine group. The redox process that explained the electron transfer of Ni (II) complex can be written as follows:

$$[{\text{Ni}}^{{ + 2}} ({\text{HL}}){\text{Cl(H}}_{{2}} {\text{O)}}_{{3}} ]^{ + } \rightleftharpoons [{\text{Ni}}^{{ + 3}} ({\text{HL}}){\text{Cl(H}}_{{2}} {\text{O)}}_{{3}} ]^{ + 2} + e^{ - }$$

#### CV study of Co (II) complex

The redox reaction of Co (II) complex at scan rate 50 mV s^−1^ exhibited two anodic peaks and three cathodic peaks as it was presented in Fig. 6. It can be investigated from the cyclic voltammograms of Co (II) complex that the oxidation peak E_pa1_ at + 0.630 V was due to the oxidation of the amine group while the oxidation peak E_pa2_ at − 0.548 V was related to the oxidation of Co (II) to Co (III). In addition, the cathodic peak E_pc1_ at − 0.855 V was corresponding to the reduction of Co (III) to Co (II) and the two reduction peaks E_pc2_ at − 0.567 V and E_pc3_ at − 0.253 V had been related to the reduction of azomethine and thione groups^60–64^.

By comparing the cyclic voltammograms of the ligand (HL) and its cobalt complex it can be observed that the oxidation peaks of Co (II) complex go to more positive value than the ligand (HL) and the reduction peaks to more negative value. This result can be interpreted as follows; the trend of oxidation peaks to more positive values may be related to the relative stability between the oxygen and sulfur atoms when bonding with cobalt ions^64^. While the trend of the reduction peaks to more negative values confirm the participation of the azomethine and thione groups in the coordination bonding. The oxidation–reduction process that can be used to explain the electron transfer of the cobalt complex can be described as follow:

$$[{\text{Co}}^{{ + 3}} ({\text{HL}}){\text{Cl}}_{{2}} {\text{(H}}_{{2}} {\text{O)}}_{{2}} ]^{ + } + {\text{e}}^{ - } \rightleftharpoons [{\text{Co}}^{{ + 2}} ({\text{HL}}){\text{Cl}}_{{2}} {\text{(H}}_{{2}} {\text{O)}}_{{2}} ]$$

#### CV study of Cd (II) complex

It can be seen from the cyclic voltammogram of Cd (II) complex at scan rate 50 mV s^−1^ in Fig. 6, that there was one ooxidation peak at + 0.761 V which it may be related to the oxidation of cadmium metal Cd (0) to cadmium anion Cd (II). And the cathodic peak at − 0.768 V which may be attributed to the reduction of Cd (II) to Cd (0) so the cathodic and the anodic reaction in this case can be written as follow:

$${\text{Cd(II) + 2e}}^{ - } \rightleftharpoons {\text{Cd(0)}}$$

$${\text{Cd(0)}} \rightleftharpoons {\text{Cd(II) + 2e}}^{ - }$$

In addition, the reduction peak at − 0.664 V may be corresponding to the reduction of azomethine or thione groups. By comparing between the position of the anodic peak of the ligand (HL) and its Cd (II) complex it can be noted that, the value of oxidation peak increased in a positive direction for the Cd (II) complex + 0.716 V more than the ligand (HL) which recorded + 0.231 V. The redox reaction that can be used to interpret the electron transfer of Cd (II) complex can be written as follow:

$$[{\text{Cd}}^{{ + 2}} ({\text{HL}})_{2} {\text{Cl}}_{{2}} ] + 2{\text{e}}^{ - } \rightleftharpoons [{\text{Cd}}^{{0}} ({\text{HL}})_{2} {\text{Cl}}_{{2}} ]^{ - 2}$$

#### The effect of changing scan rate on the ligand (HL) and its Ni (II), Co (II) and Cd (II) Complexes

**Fig. 7:**
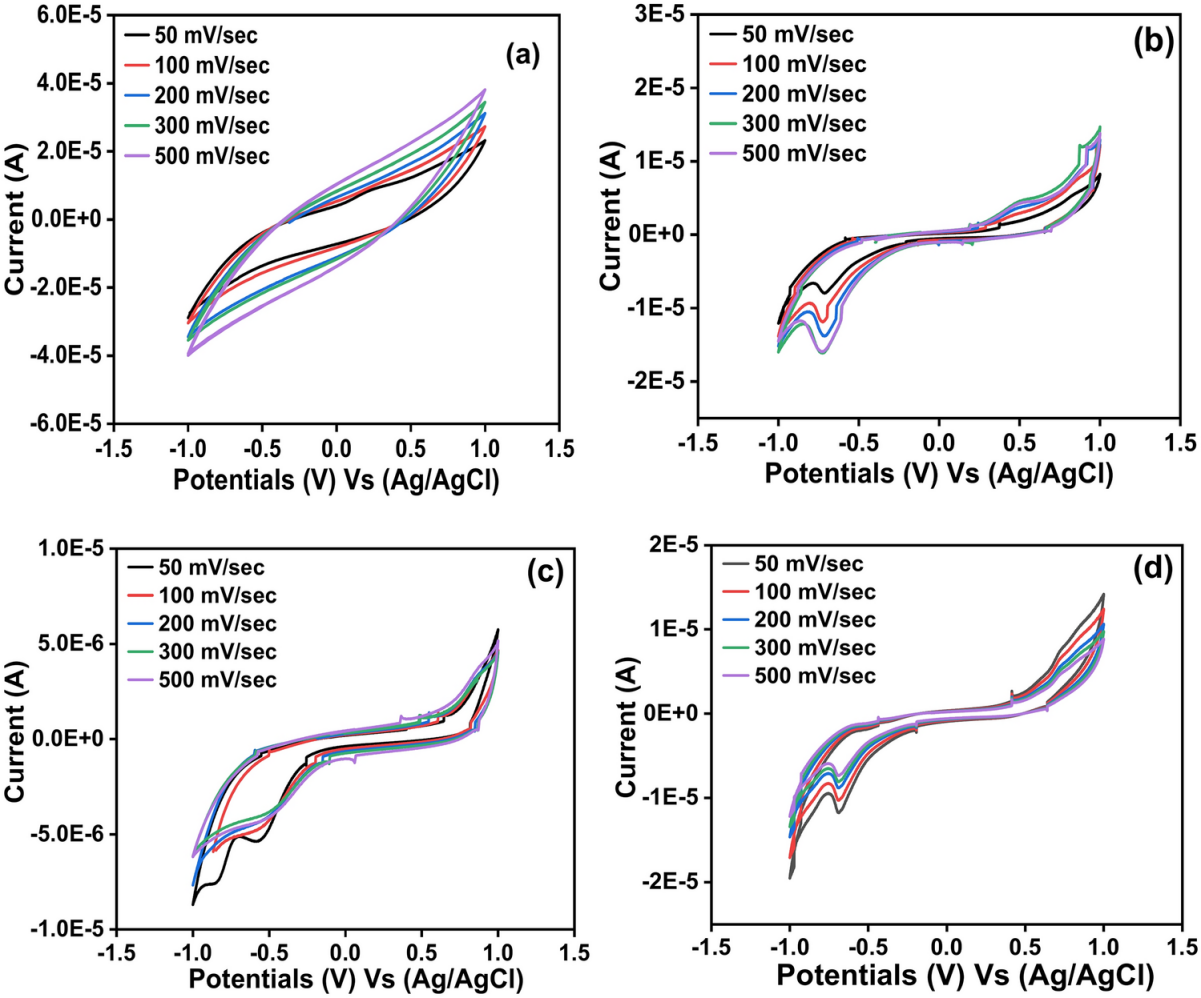
Cyclic voltammograms of the ligand HL (**a**) and its Ni (II) (**b**), Co (II) (**c**) and Cd (II) (**d**) complexes at different scan rate 50–500 mV s^−1^.

Cyclic voltammograms of the ligand (HL) and its metal complexes Ni (II), Co (II), and Cd (II) at different scan rates 50–500 mV s^−1^ had been shown in Fig. 7, as well some of their electrochemical parameters had been investigated and established in Table 3. The anodic peak potential (E_pa_), the cathodic peak potential (E_pc_), the anodic peak current (I_pa_), the cathodic peak current (I_pc_), the peak potential separation (∆E_p_), the peak current ratio (I_pa_/I_pc_) and the half-peak potentials (E_1/2_) are the electrochemical parameters which they had been deduced.

**Table 3:** Electrochemical parameters and inhibition efficiencies investigated from EIS data of the copper metal in 1.0 M HCl solution containing different concentrations of the inhibitor (HL) at 25 °C.

	Conc., M	R_s_ Ω cm^2^	R_ct_ Ω cm^2^	Q		*C*_dl_ μF cm^2−^	$IE\%$
				*Y*_o_ μΩ cm^−2^	*n*		
Blank	–	1.350	6.590	3850	0.44	17.600	–
Various conc. of the inhibitor (HL)	1 × 10^–4^	1.960	14.500	2304	0.52	9.430	54.55
	1 × 10^–5^	2.310	46.300	1145	0.64	4.760	85.76
	1 × 10^–6^	1.120	72.400	892	0.70	3.770	90.89
	1 × 10^–7^	1.160	93.200	480	0.79	2.160	92.93

**Fig. 8:**
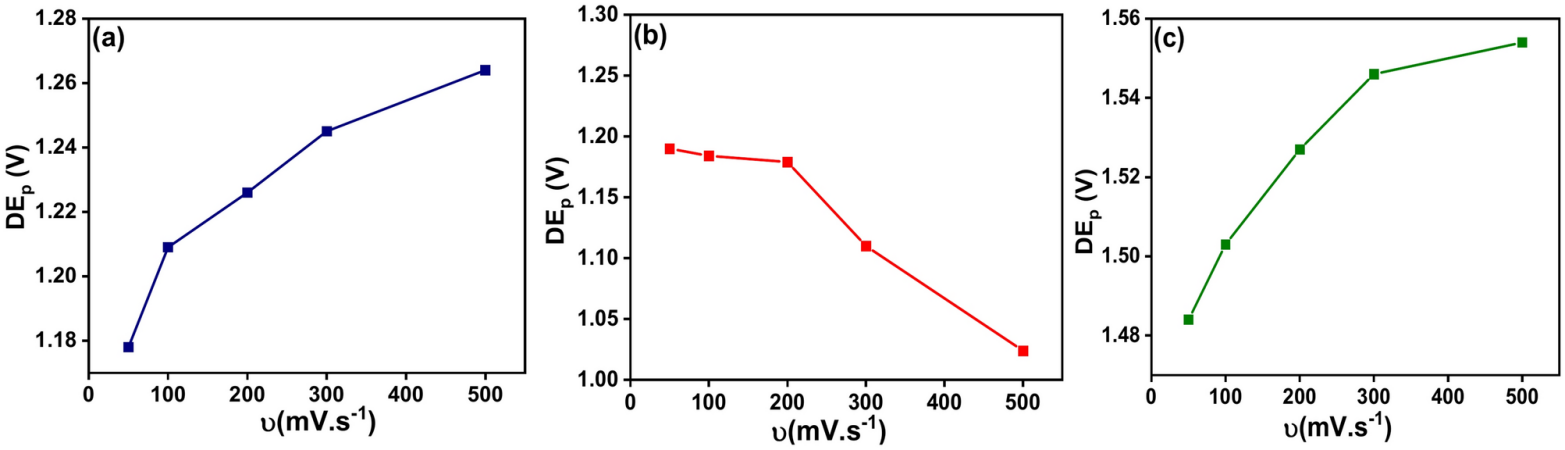
Relationship between the peak potential separation and the scan rate for the cyclic voltammograms of (**a**) Ni (II) (**b**) Co (II) and (**c**) Cd (II) complexes.

**Fig. 9:**
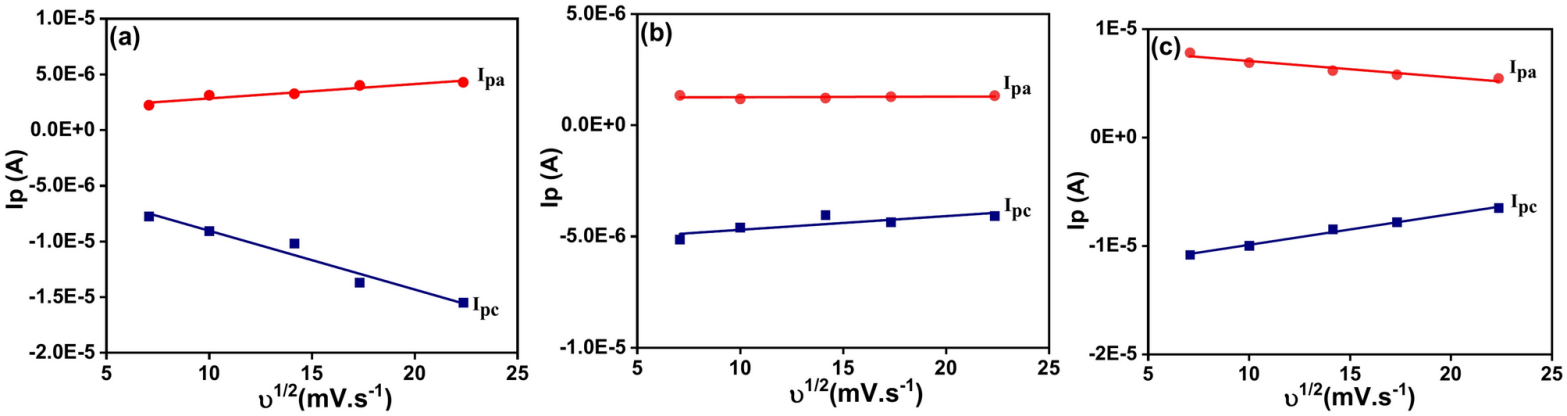
Relationship between the anodic and the cathodic current peaks versus square root of the scan rate.

It can be seen from Fig. 7, and Table 3, that, the anodic peak potential (E_pa_) and cathodic peak potential (E_pc_) of the ligand (HL) appeared only at scanning rate 50 mV s^−1^ while at scan rates higher than 50 mV s^−1^ these peaks had been disappeared. This result can be explained by the fact that, the interaction based on the electrode surface in the presence of the ligand (HL) was under adsorption controlled depending on the existence of the active sites onto the surface of the working electrode and its ability to charge transfer^66^. As well as, according to the value of the peak potential separation (∆E_p_) = (E_pa_) − (E_pc_) which recorded + 0.625 V more than + 0.059 V and the ratio of the anodic to the cathodic current peaks (I_pa_/I_pc_) was about + 0.795 less than one so this result confirms that, the redox process of the ligand (HL) was under quasi-reversible reaction. Also, the ratio of the anodic to the cathodic current peaks (I_pa_/I_pc_) for the metal complexes Ni (II), Co (II), and Cd (II) recorded value less than one and the values of the peak potential separation more than + 0.059 V as reported in Table 3. This result indicates that, the redox process for these complexes were in quasi-reversible system. In addition, from Fig. 8, which displayed the relationships between the peak potential separation and the scan rates of the metal complexes Ni (II), Co (II) and Cd (II) had been considered as an evidence that the oxidation–reduction process of these complexes under quasi-reversible reaction^67^. As well, the linear relationships between the square root of the scan rates of the metal complexes Ni(II), Co(II) and Cd(II) with both the anodic and cathodic currents as presented in Fig. 9, was considered as an evidence that the reaction occurred at the working electrode under diffusion control^68^ for the voltammograms of (a) Ni (II) (b) Co (II) and (c) Cd (II) complexes.

## Antibacterial study

**Fig. 10:**
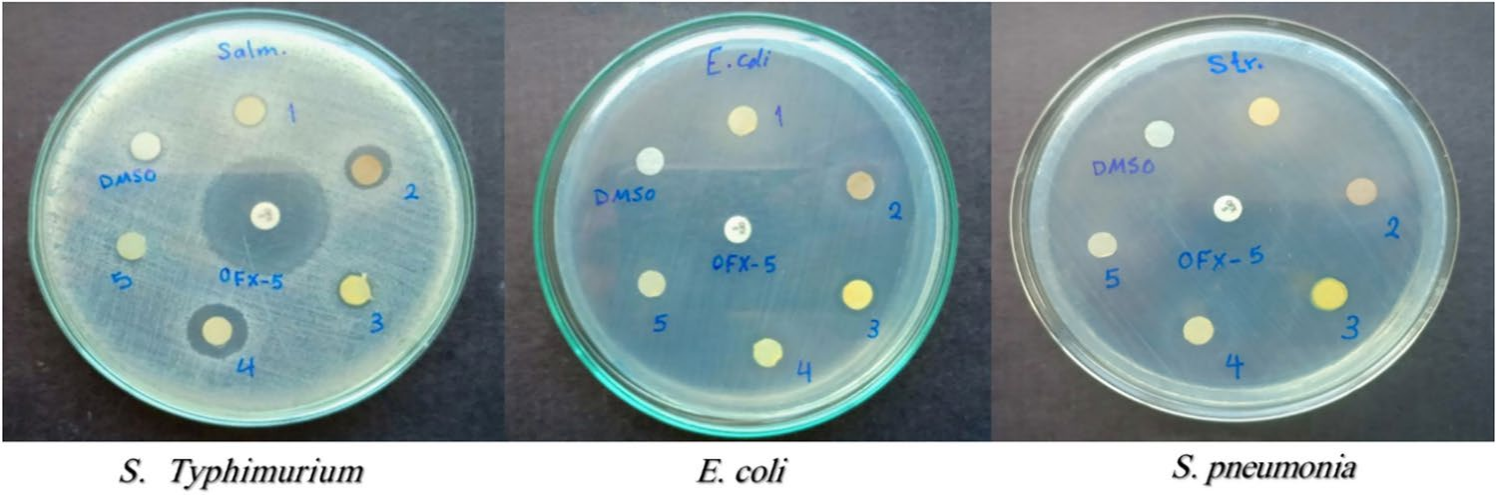
Zone of inhibition of *S. Typhimurium, E. coli* and *S. pneumoni* for the inhibitor (HL) and its Ni (II), Co (II) and Cd (II) complexes.

The antibacterial activity of the prepared compounds against *S. aureus* and *S. pneumoniae* as representatives for gram- positive bacteria o and *E. coli* and *S. Typhimurium* as representatives for gram- negative bacteria was measured by measuring the zones of inhibition of bacterial growth using disc diffusion method. The resulting inhibition zones were compared with the inhibition zone of the standard antibiotic Ofloxacin discs (OFX-5), as well as the inhibition zone caused by DMSO Table 4, and Fig. 10. The Co (II) and Cd (II), complexes showed the best antibacterial activity against *S. pneumonia* with inhibition zones of (1.2 ± 0.2 cm), and (1.1 ± 0.2 cm), respectively as compared to (3.0 ± 0.1 cm), for ofloxacin; and against *S. Typhimurium* with inhibition zones of (1.0 ± 0.0 cm), and (1.3 ± 0.2 cm), respectively as compared to (3.5 ± 0.1 cm), for ofloxacin. Meanwhile, all compounds did not show any antibacterial activity against *S. aureus*. The Co (II) and Cd (II), complexes also exhibited antibacterial activity against *E. col*i with inhibition zones of (1.0 ± 0.1 cm), and (1.0 ± 0.2 cm), respectively as compared to (4.0 ± 0.1 cm), for ofloxacin, Table 5.

**Table 4:** The investigated electrochemical parameters from the cyclic voltammograms of 0.005 M solution of the ligand (HL) and its Ni(II), Co(II) and Cd(II) complexes with 0.05 M solution of TBAP in DMSO at different scan rates 50–500 mV s^−1^.

Compounds	Different scan rates	(Ep_a_)	(Ep_c_)	(Ip_a_)	(Ip_c_)	(Ip_a_/Ip_c_)	(∆E_p_)	(E_1/2_)
	50	+ 0.231 + 0.041	− 0.394	9.06 × 10^–6^ 4.4 × 10^–6^	− 1.14 × 10^–5^	− 0.795	+ 0.625	− 0.082
Ligand (HL)	100	–	–	–	–	–	–	–
	200	–	–	–	–	–	–	–
	300	–	–	–	–	–	–	–
	500	–	–	–	–	–	–	–
Ni (II) complex	50	+ 0.471	− 0.707	2.23 × 10^–6^	− 7.75 × 10^–6^	− 0.28	1.178	− 0.118
	100	+ 0.495	− 0.714	3.12 × 10^–6^	− 9.08 × 10^–6^	− 0.344	1.209	− 0.109
	200	+ 0.507	− 0.719	3.27 × 10^–6^	− 1.02 × 10^–5^	− 0.321	1.226	− 0.106
	300	+ 0.513	− 0.732	4.01 × 10^–6^	− 1.37 × 10^–5^	− 0.292	1.245	− 0.109
	500	+ 0.520	− 0.744	4.29 × 10^–6^	1.55 × 10^–5^	− 0.277	1.264	− 0.112
Co (II) complex First peak	50	0.630	− 0.560	1.13 × 10^–6^	− 5.12 × 10^–6^	− 0.221	1.190	0.035
	100	0.606	− 0.578	1.19 × 10^–6^	− 4.68 × 10^–6^	− 0.254	1.184	0.014
	200	0.583	− 0.596	1.22 × 10^–6^	− 4.43 × 10^–6^	− 0.275	1.179	− 0.006
	300	0.501	− 0.609	1.28 × 10^–6^	− 4.31 × 10^–6^	− 0.297	1.110	− 0.054
	500	0.397	− 0.627	1.33 × 10^–6^	− 4.07 × 10^–6^	− 0.327	1.024	− 0.115
Co (II) complex Second peak	50	− 0.548	− 0.253	− 6.09 × 10^–7^	− 7.99 × 10^–7^	0.762	− 0.295	− 0.401
	100	− 0.510	− 0.198	− 7.34 × 10^–7^	− 9.83 × 10^–7^	0.747	− 0.312	− 0.354
	200	− 0.596	− 0.148	− 5.50 × 10^–7^	− 1.04 × 10^–6^	0.529	− 0.448	− 0.372
	300	− 0.590	− 0.105	− 6.74 × 10^–7^	− 1.16 × 10^–6^	0.581	− 0.485	− 0.347
	500	− 0.576	+ 0.061	− 6.25 × 10^–7^	− 1.29 × 10^–6^	0.472	− 0.637	− 0.257
Co (II) complex Third peak	50	–	–	–	–	–	–	–
	100	+ 0.822	–	9.96 × 10^–7^	–	–	–	–
	200	+ 0.839	–	9.31 × 10^–7^	–	–	–	–
	300	+ 0.876	–	8.72 × 10^–7^	–	–	–	–
	500	+ 0.889	–	7.74 × 10^–7^	–	–	–	–
Cd(II) complex first peak	50	+ 0.716	− 0.768	7.61 × 10^–6^	− 9.16 × 10^–6^	− 0.831	+ 1.484	− 0.026
	100	+ 0.728	− 0.775	6.89 × 10^–6^	− 8.35 × 10^–6^	− 0.825	+ 1.503	− 0.024
	200	+ 0.746	− 0.781	6.16 × 10^–6^	− 6.80 × 10^–6^	− 0.906	+ 1.527	− 0.018
	300	+ 0.759	− 0.787	5.78 × 10^–6^	− 6.48 × 10^–6^	− 0.892	+ 1.546	− 0.014
	500	+ 0.765	− 0.789	5.44 × 10^–6^	− 5.79 × 10^–6^	− 0.939	+ 1.554	− 0.012
Cd(II) complex second peak	50	–	− 0.707	–	− 10.82 × 10^–6^	–	–	–
	100	–	− 0.714	–	− 9.98 × 10^–6^	–	–	–
	200	–	− 0.719	–	− 8.65 × 10^–6^	–	–	–
	300	–	− 0.732	–	− 7.82 × 10^–6^	–	–	–
	500	–	− 0.744	–	− 6.50 × 10^–6^	–	–	–

## Surface characterization

**Fig. 11:**
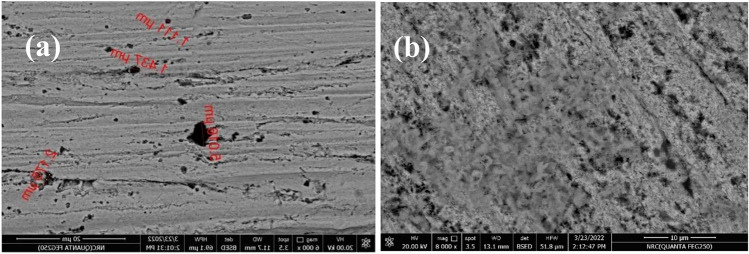
SEM micrographs of the copper metal (**a**) copper sample in the solution 1.0 M HCl without the inhibitor (HL) (**b**) copper sample in presence of the inhibitor (HL) in the solution 1.0 M HCl at 25 °C.

The surface and constituents of the copper samples had been characterized by scanning electron microscopy SEM and energy dispersive x-ray EDX. Figure 11a, b presents a morphological image for the copper surface after the potentio-dynamic polarization had been carried out in the solution 1.0 M HCl without and with the optimum concentration 1 × 10^−7^ M of the inhibitor (HL). Figure 11a shows the extent of damage caused onto the surface of the copper sample after it was exposed to an aggressive medium 1.0 M HCl without the inhibitor (HL). Therefore, the dangerous growth of localized attack (pits) on the sample surface can be observed. After adding the ideal concentration of inhibitor (HL) a protective layer was formed on the surface of the sample as shown in Fig. 11b.

**Fig. 12:**
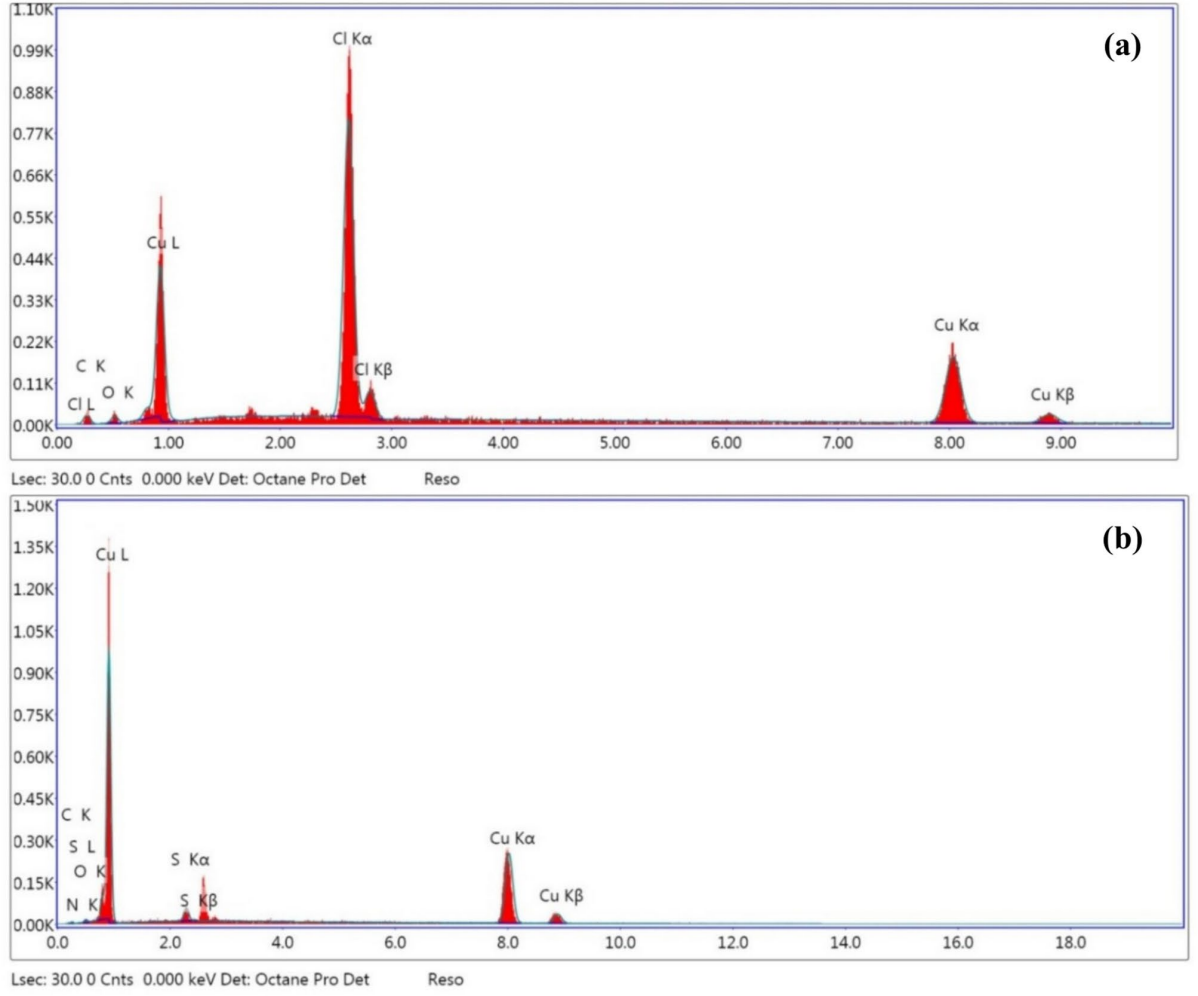
EDX analysis of the copper metal (**a**) copper sample in the solution 1.0 M HCl without the inhibitor (HL) (**b**) copper sample in presence of the inhibitor (HL) in the solution 1.0 M HCl at 25 °C.

This result indicates that the inhibitor (HL) can improve the inhibition efficiency of the copper metal against the action of the corrosive media. Energy dispersive x-ray (EDX) analyses of the copper sample in presence of the inhibitor (HL) shows and confirms that, the inhibitor molecules had been adsorbed on the copper surface. This result was illustrated through the appearance of nitrogen and sulfur peaks while the chloride peaks disappeared. This result indicates that, a protective layer of the inhibitor (HL) was formed onto the copper surface which led to impede the penetration of the chloride ions to the surface of the metal and thus prevented the dissolution of the copper metal. This result was presented and tabulated in Fig. 12a, b, and Table 5.

**Table 5:** Antibacterial activity of the studied compounds.

Sample no. on plate	Zone of Inhibition/cm			
	*S. aureus*	*S. pneumonia*	*E.coli*	*S. Typhimurium*
Ni (II) complex	–	–	–	0.7 ± 0.1
Co (II) complex	–	1.2 ± 0.2	1.0 ± 0.1	1.0 ± 0.0
Cd(II) complex	–	1.1 ± 0.2	1.0 ± 0.2	1.3 ± 0.2
Ligand (HL)	–	–	–	0.6 ± 0.1
OFX-5		3.0 ± 0.1	4.0 ± 0.1	3.5 ± 0.1
DMSO	–	–	–	–

## DFT analysis

### Geometry optimization

**Fig. 13:**
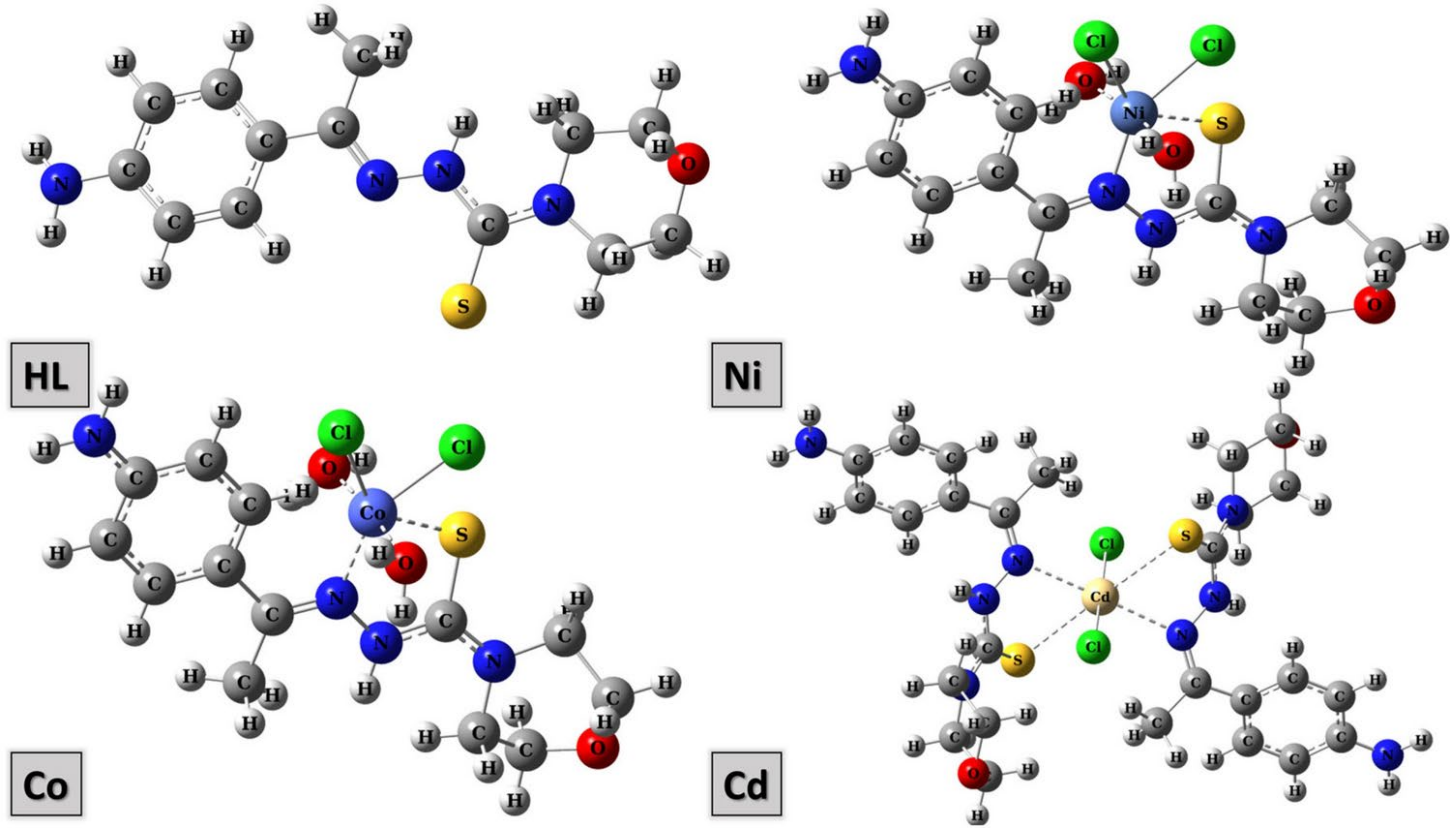
Optimized molecular structure of HL. Ni (II), Co(II) and Cd(II) complexes.

The optimized geometry of the ligand (HL) and the studied complexes had been studied and their lowest energy configuration is shown in Fig. 13, and Table 6. The energies of the complexes range from 1700–1800 HF. The polarity of ligand decreases after co-ordination with metals to form complexes which can be observed from the magnitude of their dipole moments. Cd(II) complex had the highest dipole momentum followed by Ni (II) then Co(II).

**Table 6:** Constituent elements of the copper sample after potentio-dynamic polarization process in the solution 1.0 M HCl in the absence and presence of the inhibitor (HL).

Test	Sample	Elements weight %					
		Cu	N	Cl	S	O	C
Without inhibitor (HL)	Copper metal	47.86	–	32.44	–	3.11	16.59
with inhibitor (HL)		92.28	0.27	–	2.84	1.23	3.38

### Molecular orbital calculations

**Fig. 14:**
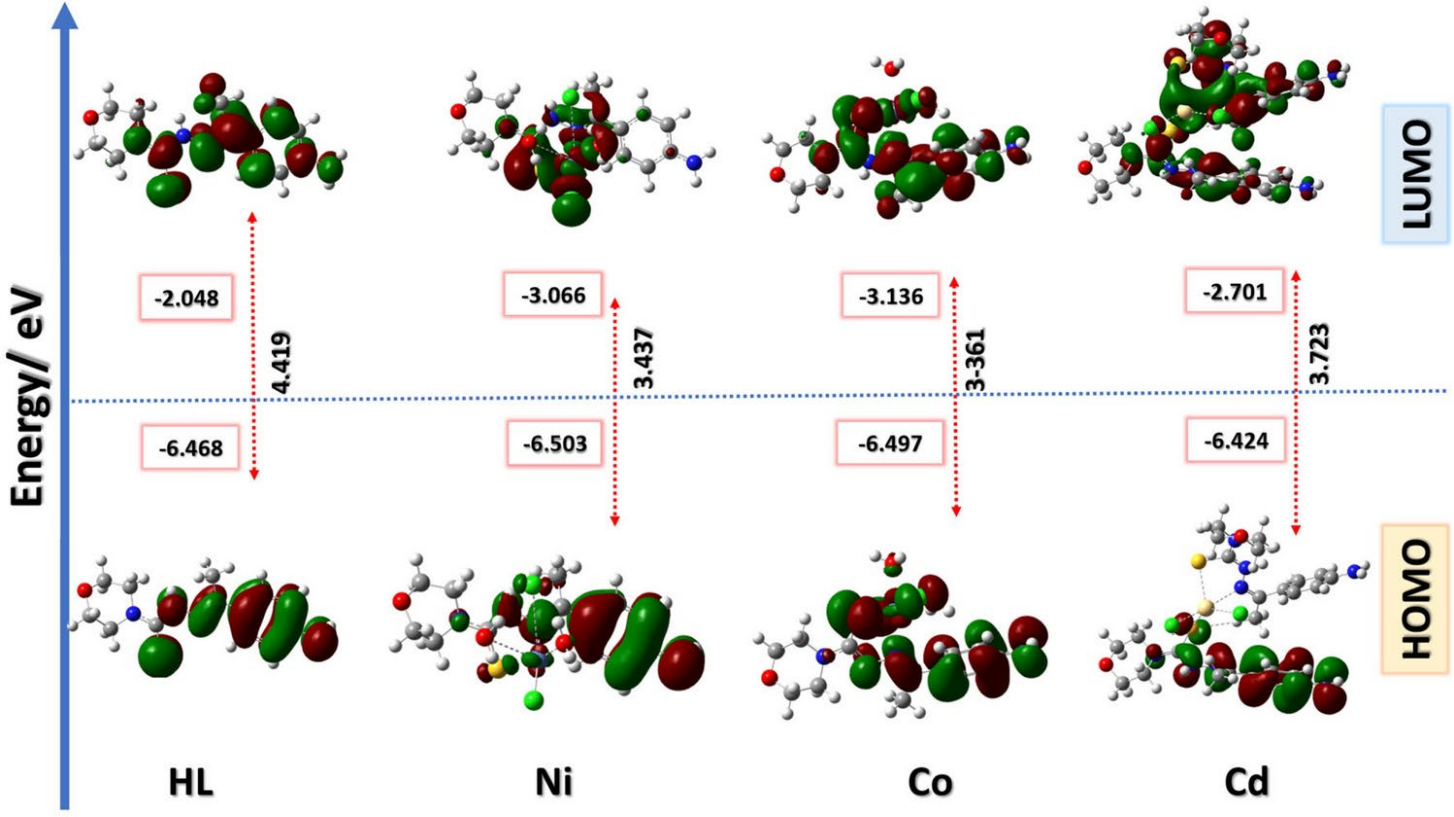
HOMO and LUMO frontier molecular orbitals along with their energies diagram for the ligand H_4_L and their complexes calculated in vacuum at CAM-B3LYP/LAN2DZ.

Frontier molecular orbital shapes studied in the ground state were illustrated in Fig. 14, along with molecular energy diagram. The global reactivity indexes, such as the ionization potential (I), electron affinity (A), absolute electronegativity (*X*), absolute hardness (η), and softness (S), for the ligand and the studied complexes were calculated at the same computational level, and the results were presented in Table 6. The band gap between HOMO and LUMO orbitals represents the amount of charge transfer interaction that could take place between the ligand and metal after complexation. The band gab reflects how much the complex will be biologically active^30^. The molecule with the smaller band gab is associated with high chemical reactivity and defined as a soft molecule^69,70^.

Molecular orbital analysis was done to explain structure activity relationship (SAR) of the ligand (HL) and their chelates (Ni(II), Co(II) and Cd(II)) that were under study^66,67,71^. In this sense, a molecule’s biological activity and toxicity, based on a theoretical density functional theory, global and local reactivity descriptors had been created. Furthermore, frontier molecular orbitals had been used to assess the global reactivity indices of the substances under study. The HOMO and LUMO (highest occupied molecular orbitals and lowest unoccupied molecular orbitals) are referred to electron donor and acceptor, respectively. The different in energy between HOMO and LUMO (band gab or ΔE) is responsible for kinetic stability and chemical reactivity of the molecule, the high or low band gab are referred to how hard or soft the molecules, in addition the smaller the energy value means the high biological efficiency of the molecule^68–71^ The HOMOs and LUMOs of the (HL) are substantially delocalized over thiosemicarbazone ligand Fig. 14. The ligand (HL) and its Cd(II) complex have the lowest values of band gap, which allows them to be the softness molecules among all studied complexes and the highest predicted biologically active molecules. For Cd(II) complex, the HOMO is mainly located above one thiosemicarbazone, meanwhile the LUMO is delocalized over the both chelated ligands, and it follows by Ni(II) and Co(II) complexes in the band gab value and the softness property. For Ni (II) and Co(II) complexes, the HOMOs were mainly located over thiosemicarbazone part, Meanwhile, the LUMOs were mainly distributed over the metal coordination part with some contribution on the ligand unit. To understand this part, Fig. 14, explains the energy level diagram for ligand (HL) and the studied complexes. The HOMO and LUMO of the ligand were destabilized by the addition of the metals in case of Ni(II) and Co(II) complexes, however, the energies are dependent on the properties of the specific metal and mode of coordination.

The energies obtained via DFT calculations were good support to explain the biological results as the ligand and Cd(II) have highest potency in comparison to other chelated complexes.

## Molecular docking studies

**Fig. 15:**
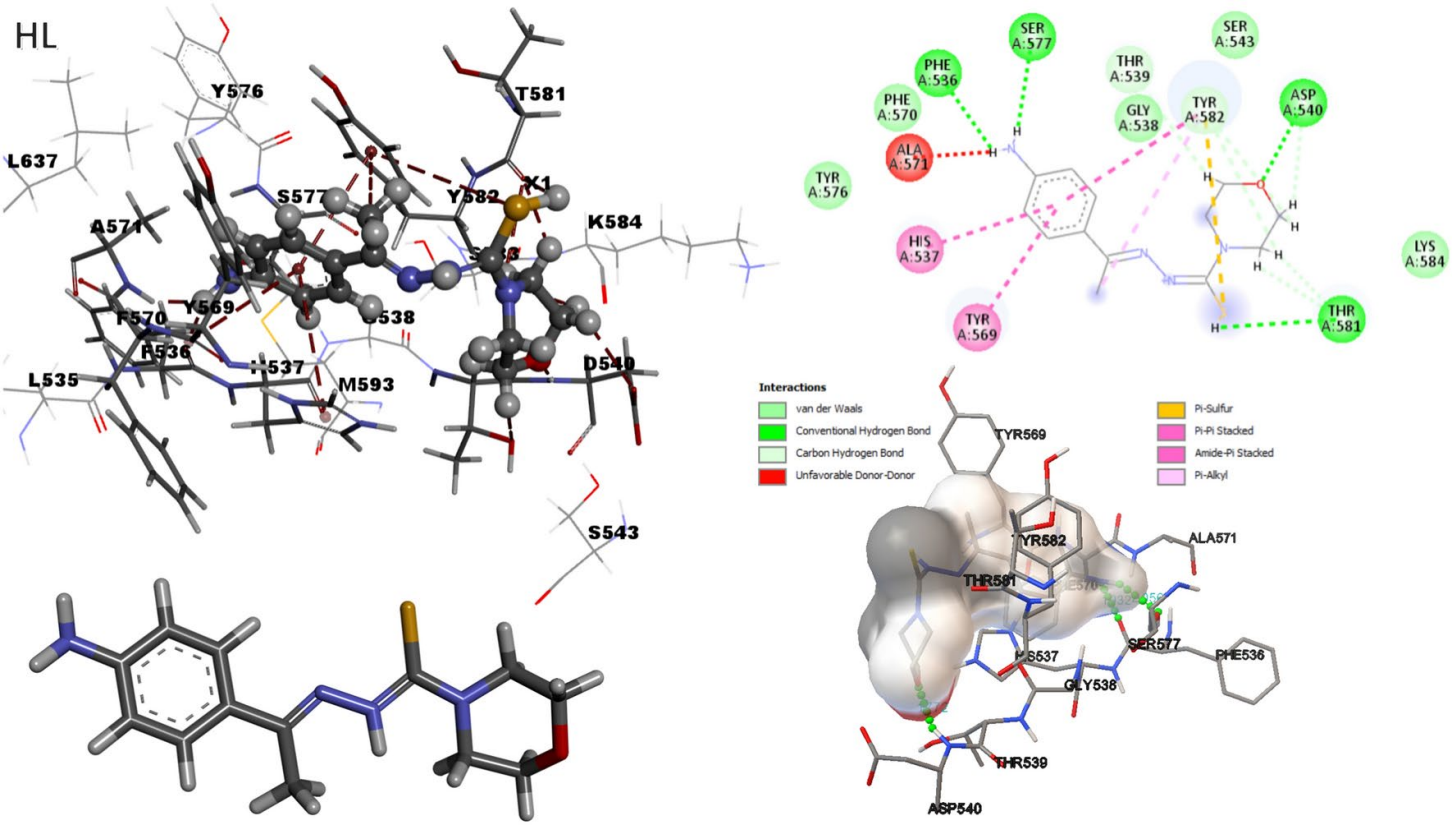
2D and 3D docking interactions of ligand (HL) and the Ni(II), Co(II) and Cd(II) complexes in the active pocket of ribosyl transferase (PDB ID: 3GEY) visualized with discovery studio software.

The studied complexes were derivatives from thiosemicarbazide which are well known for their biological efficiencies. Molecular docking techniques were used to explore their mechanism of action as antimicrobial agents. Molecular docking visualization was performed to predict the interaction binding mode of active inhibitors^72–74^ with the crystal structure of ribosyltransferase (PDB ID: **3GEY**)^75^. The docking analysis results were shown in Table 7. The docking results reveal that both ligand and Ni(II) complex had the highest potential due to high interaction in the active site of the protein and the lowest docking energy compared with the new target compounds. The docking energies for all compounds were in the range from (-6.3: -8.9) Kcal\mol. The Ki explains the potency of inhibitors related to their concentration requirement for half-maximal inhibition. In addition to measuring some important values such as intermolecular and internal interactions surrounding the complexed structure to choose the best active conformer. The best docked pose was selected considering its intermolecular, internal, and inhibition constant (Ki) of the conformational structure. K_i_ expresses the potency of inhibitor related to its concentration requirement for half-maximal inhibition, all these parameters were tabulated in Table 7. All the compounds were docked in the active site of **3GEY** protein efficiently. The binding mode and 3D interaction of the ligand was shown in Fig. 15. The ligand showed three hydrogen bonds with the active site residues Phe536, Asp540 and Ser577 meanwhile, Co(II) and Cd(II) have only one H bond with Asp540 and Tyr41. Figure 15. shows that the best conformation of the docked ligand is shown as a graphical 3D and 2D representations with surrounding residues interaction. There are several docking interactions represented in conventional H-bond, carbon–hydrogen bond, Van der Waals (vdW), and π-lone pair interactions (Table 8). These interactions mainly help in the inhibition efficiency of the synthesized ligand.

**Table 7:** Theoretical energy calculations and dipole moment of the studied compounds and their interaction products.

ID	E_total_ (HF)	Dipole momentum	Energy (eV)			I	A	η	S	µ	X
			E_HOMO_	E_LUMO_	ΔE						
HL	− 916.456	5.868	− 6.468	− 2.048	4.419	6.468	2.048	− 2.210	− 0.453	− 1.524	4.258
Ni	− 1849.822	9.124891	− 6.503	− 3.066	3.437	6.503	3.066	− 1.719	− 0.582	− 2.033	4.785
Co	− 1849.831	8.541	− 6.497	− 3.136	3.361	6.497	3.136	− 1.680	− 0.595	− 2.068	4.817
Cd	− 1744.539	12.458	− 6.424	− 2.701	3.723	6.424	2.701	− 1.862	− 0.537	− 1.851	4.563

**Table 8:** Molecular docking scores for all compounds, binding energies, and Protein–Ligand interaction of the synthesized molecules incise the active site of ribosyltransferase (PDB ID: **3GEY**).

No	Binding energy kcal/mol)	Intermolecular energy	Internal energy	Inhibition constant	Protein–Ligand interaction and bond distance Å
HL	− 6.29	− 8.58	− 7.78	24.48 µM	O-PHE536 1.932 NH-ASP540 1.772 O-SER577 2.056
Ni	− 8.91	− 9.81	− 0.93	294.15 µM	O-PHE536 1.256 NH-ASP540 2.032 O-SER577 1.256 O-SER577 1.235
Co	− 7.74	− 8.93	− 0.6	2.12 µM	NH-ASP540 2.109
Cd	− 7.24	− 6.03	− 1.35	7.76 µM	NH-TYR41 1.761

## Conclusions

1. In the present study, the thiosemicarbazone ligand (HL) was examined to be used as a corrosion inhibitor for copper metal when exposed to aggressive media such as hydrochloric acid which was used in this work.
2. The electrochemical measurements applied in this study contains; open circuit potential (OCP) potentio-dynamic polarization (PDP) and electrochemical impedance spectroscopy (EIS).
3. The percentage of the inhibition efficiency was calculated after the electrochemical measurements had been carried out which recorded a percentage value about 94.66% and 92.93% for (PDP) and (EIS) methods respectively.
4. According to the above result, the ligand (HL) is considered as a new corrosion inhibitor for metals.
5. The redox reactions that, occurred onto the surface of the ligand (HL) and its metal complexes Ni(II), Co(II) and Cd(II) had been determined by using cyclic voltammatery technique.
6. The results deduced after the cyclic voltammatery technique was carried out proved that, the system of the oxidation–reduction process of the ligand (HL) and its metal complexes Ni(II), Co(II) and Cd(II), had been under quasi-reversible and diffusion control.
7. To know the redox reactions of the system is very important in our life for example, rusting of iron metal, combustion of fuels such as gasoline or wood, respiration in living organisms, corrosion of metals and photosynthesis in plants
8. The ligand (HL) and its metal complexes Ni(II), Co(II) and Cd(II) exhibited good antibacterial activity against *S. pneumonia*, *S. Typhimurium* and *E. coli*, but the Co(II) and Cd(II) complexes showed the best result.
9. Based on their antimicrobial results, DFT studies have been done to explain the electronic properties of all the compounds studied.
10. The DFT results revealed that both ligand (HL) and Ni(II) complex have the lowest band gap and thus, nearby biological efficiencies. Moreover, the studied descriptors helped us to understand the activities of all the studied complexes based on their electronic configuration.
11. Further, molecular docking study had been performed along with antimicrobial study; the results spot a clear explanation of the compound interaction sites.
12. The modelling results concluded that, the interaction of thiosemicabazone ligand (HL), with the amino acid of the active site increases their activity; specially for the ligand (HL), followed by Ni(II), complex configuration as a promising antimicrobial agent so, it can be used for treating a selected range of microbial infections.

## Supplementary Information

Supplementary Information.

## Data Availability

The datasets used and/or analysed during the current study available from the corresponding author on reasonable request.
