# Supplementary material for: Exploring corrosion behavior, antimicrobial evaluation, molecular docking and DFT calculation of thiosemicarbazone ligand and its metal complexes
Source: Sci Rep. 2025 May 13;15:16577. doi: 10.1038/s41598-025-98580-1 (PMC12075617; doi:10.1038/s41598-025-98580-1)
Supplement: Supplementary file 1 — Supplementary Information. [file 41598_2025_98580_MOESM1_ESM.docx]

**Supporting information**

**Exploring corrosion behavior, antimicrobial evaluation, molecular docking and DFT calculation of thiosemicarbazone ligand and its metal complexes**

Howida S. Mandour ^a*^, Lobna A. Khorshed ^a^, Amr M. Abdou ^b^ , Basma Ghazal ^c*^

^a^ *Physical Chemistry Department, National Research Centre, 33 El Bohoth St., Dokki, P.O. 12622, Giza, Egypt*.

*^b^ Microbiology and Immunology Department, National Research Centre, 33 El Bohoth St., Dokki, P.O. 12622, Giza, Egypt.*

^c^Department of Organometallic and Organometalloid Chemistry Division, National Research Centre, Giza, Egypt

* Corresponding author [Hmandour77@gmail.com](mailto:Hmandour77@gmail.com)

basmaghazal@gmail.com

# Results and Discussion

The characterizations had been used to prove the suggested structures of the ligand (HL) and its metal complexes which had been studied in the current work (Ni (II), Co (II) and Cd) included (analytical, spectral, magnetic moment and thermal analyses).

Elemental analysis and physical date of the ligand (HL) and its (Ni (II), Co (II) and Cd complexes

**Table (S1)** illustrates the colors, elemental analyses, chemical formulae, melting points and molar conductivity values of the prepared compounds. Metal complexes with different stoichiometry; 1:1 and 1:2 (M: L) arise from the reactions of the thiosemicarbazone ligand (L) with different metal salts. All obtained metal complexes are stable, non-hygroscopic and freely soluble in DMF and DMSO

**Table.S1.** Analytical and physical data of the thiosemicarbazone ligand (HL) and its metal complexes

| No | Compound | F.W | Elemental analysis Found/(calc)% | | | | | | M.P.  (°C) | *Λ*  (Ω^-1^cm^2^mol^-1^) |
| --- | --- | --- | --- | --- | --- | --- | --- | --- | --- | --- |
|  |  | Color | C | H | N | S | Cl/Br | M |  |  |
|  | HL  C_13_H_18_N_4_SO | 278.38  Yellow | 55.67  (56.09) | 6.67  (6.52) | 19.95  (20.13 | 11.61  (11.52) | -  - | -  - | 131 | - |
| 1 | [Co(HL)Cl_2_(H_2_O)_2_].2.75H_2_O  C_13_H_22.5_N_4_SO_5.75_CoCl_2_ | 493.88  Brown | 31.28  (31.62) | 5.77  (5.61) | 11.88  (11.34) | 7.11  (6.49) | 14.73  (14.38) | 12.69  (11.93) | 189 | 23 |
| 2 | [Ni(HL)Cl(H_2_O)_3_].Cl.3.5H_2_O  C_13_H_31_N_4_SO_7.5_NiCl_2_ | 525.12  Yellowish brown | 30.17  (29.73) | 5.57  (5.61) | 10.79  (10.67) | 6.27  (6.09) | 13.88  (13.52) | 11.64  (11.18) | 223 | 76 |
| 3 | [Cd(HL)_2_Cl_2_].4EtOH.4H_2_O  C_34_H_68_N_8_S_2_O_10_CdCl_2_ | 996.51  Yellow | 41.07  (40.98) | 6.43  (6.88) | 11.33  (11.24) | 6.91  (6.44) | 6.61  (7.12) | 10.82  (11.28) | 165 | 3 |

## Mass Spectrum

**Figure (S1),** the (FAB-MS) of the thiosemicarbazone ligand (HL) displays a molecular ion peak at m/z ¼ 278 amu, corresponding to the calculated theoretical molecular weight. This value proposed the chemical formula (C13H18N4SO) which is based on the elemental analyses data of the ligand


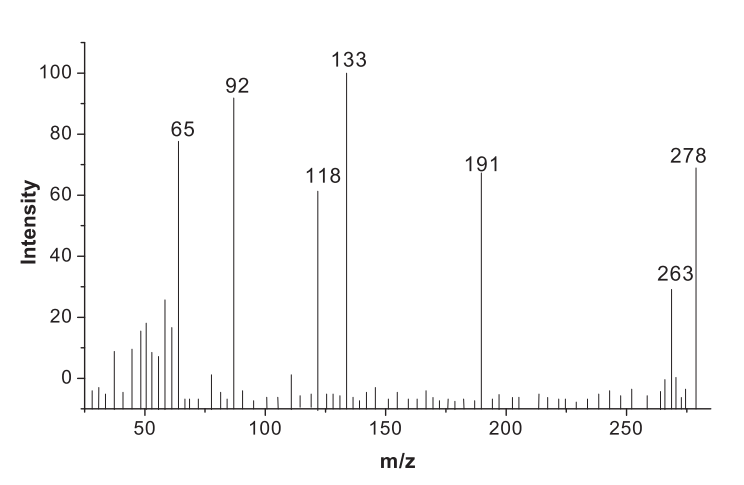


**Fig. S1.** Mass spectrum of the thiosemicarbazone ligand (HL)

## ^1^H NMR of ligand (HL) and Cd(II) complex

The ^1^ H NMR spectrum of the ligand displays two signals at d 10.47 and 9.87 ppm which enriches the basis of *Z*-*E* stereoisomerism. It can be seen from **Table (S2),** that cadmium complex exhibit only the NH signal of *E* form at d 10.11 and 9.92 ppm, respectively, established that those complexes are separated in *E* isomer only.

**Table.S2.** 1H NMR date of the ligand and its cadmium complex

| Ligand (HL) | [Cd(HL)_2_Cl_2_].4EtOH.4H_2_O complex | Assignment |
| --- | --- | --- |
| 10.47 | - | -NH (*Z*- form) |
| 9.87 | 9.92 | -NH (*E*- form) |
| 6.53 – 7.99 | 6.52 – 7.99 | aromatic ring protons |
| 5.44 | 5.49 | -NH_2_ protons |
| - | 3.89 | solvent protons |
| 3.07 – 3.82 | 3.07 – 3.75 | -CH_2_ protons |
| 2.15 – 2.48 | 2.15 – 2.47 | -CH_3_ protons |

## IR spectra of the thiosemicarbazone ligand and its Co(II) complex

The comparison between the IR spectra of the ligand and prepared metal complexes gives an insight into the binding behavior of the ligand. For example, the FT-IR spectra of the thiosemicarbazone ligand and its Co(II) complex had been shown in **Fig.S2.** The most prominent IR spectral features of the ligand and its Ni (II), Co (II) and Cd complexes are tabulated in **Table** **(S3)**.

**Table. S3.** Infrared spectral bands (cm^-1^) of the thiosemicarbazone ligand and its Ni (II), Co (II) and Cd complexes.

| No | Compound | *υ*(OH)+ *υ*(NH_2_)/ *υ*(NH) | *υ* (C=N)+δ(NH_2_) | Thioamide bands | | | | *υ*(N-NH) | γ(C=S) | γ (NH) | υ(M-O) | υ(M-N) |
| --- | --- | --- | --- | --- | --- | --- | --- | --- | --- | --- | --- | --- |
|  |  |  |  | I | II | III | IV |  |  |  |  |  |
|  | HL | 3445(s.b)  3398(m)  3375(s) | 1620(s) | 1533(s) | 1291(s) | 1223(s) | 904(m) | 1036(s) | 505(m) | 694(m) | - | - |
| 1 | **[Co(HL)Cl_2_(H_2_O)_2_].2.75H_2_O** | 3424(s.b) | 1618(s) | 1573(w) | 1276(s) | 1232(s) | 887(s) | 1031(m) | 546(w) | 690(w) | 486(w) | 456(w) |
| 2 | **[Ni(HL)Cl(H_2_O)_3_].Cl.3.5H_2_O** | 3419(s.b) | 1633(s) | 1573(m) | 1276(s) | 1248(m) | 891(m) | 1025(s) | 546(w) | 680(m) | 483(w) | 425(w) |
| 3 | **[Cd(HL)_2_Cl_2_].4EtOH.4H_2_O** | 3473(w)  3446(w) | 1622(s) | 1594(s)  1546(vw) | 1271(s) | 1231(s) | 883(s) | 1030(m) | 513(m) | 683(w) | 481(vw) | 424(w) |

vw: very weak, w: weak, m: medium, s: strong, b: broad, sh: shoulder, sp: splitted


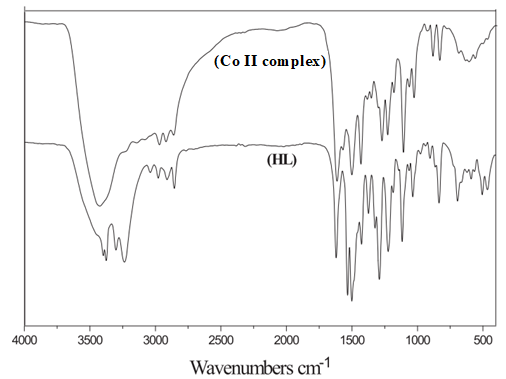


**Fig. S2.** IR spectra of the thiosemicarbazone ligand and its Co(II) complex

## Electronic spectra (UV-VIS) and magnetic moments of the thiosemicarbazone ligand (HL) and its Ni (II), Co(II) and Cd(II) complexes

The electronic absorption spectra and the magnetic moment values are important techniques for elucidating the stereochemistry of the metal complexes. **Table (S4),** displayed the magnetic moment values and the electronic spectral data of the ligand (HL) and its Ni(II), Co(II) and Cd(II) complexes.

**Table. S4.** Electronic spectral data and magnetic moment values of the ligand and its metal complexes.

| No | Compound | Electronic spectra bands (nm) | Assignment | μ_eff._ (B.M.)* |
| --- | --- | --- | --- | --- |
|  | HL | 320.367  265 | π→π*  π→π* | - |
| 1 | **[Co(HL)Cl_2_(H_2_O)_2_].2.75H_2_O** | 687  586  434  269, 334 | ^4^T_1g_(F) → ^4^A_2g_(P)  ^4^T_1g_(F) → ^4^A_2g_(P)  LMCT  Intraligand transition | 5.07 |
| 2 | **[Ni(HL)Cl(H_2_O)_3_].Cl.3.5H_2_O** | 681  518  436  273, 320 | ^3^A_2g_(F) → ^3^T_1g_(F)  ^3^A_2g_(F) → ^3^T_1g_(P)  LMCT  Intraligand transition | 3.56 |
| 3 | **[Cd(HL)_2_Cl_2_].4EtOH.4H_2_O** | 440  271, 345 | LMCT  Intraligand transition | Diamagnetic |

*: (per metal ion).

1. **Thermal analysis of the ligand and its Ni (II), Co(II) and Cd(II) complexes**

The results achieved from the thermal analyses (TG, DTG) of the ligand and its metal complexes are listed in **Table (S5) and Fig (S3).** The data show that all metal complexes have higher thermal stability than that of the free ligand. **Scheme (S1)** displayed the thermal decomposition of the ligand (HL) its Co(II) complex as an example.


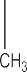

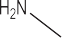

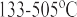

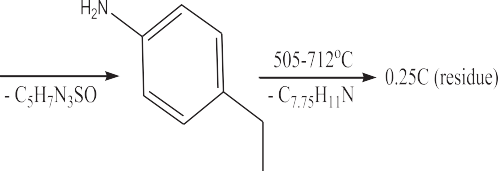


**(a)**

**(a)** The proposed thermal decomposition mechanism for the ligand (HL)


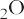

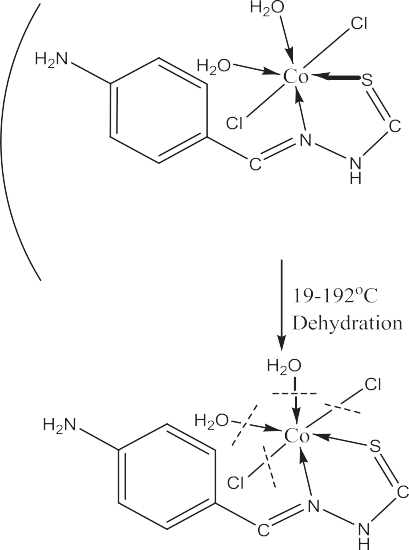

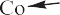

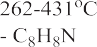

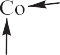

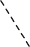


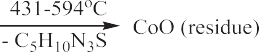


**(b)**

**(b) Thermal decomposition mechanism for [Co(HL)Cl_2_(H_2_O)_2_].2.75H_2_O complex**

**Scheme (S1)**


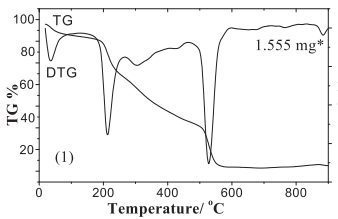

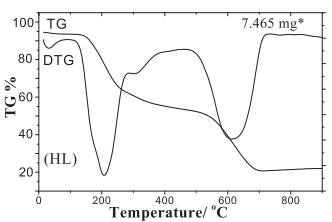


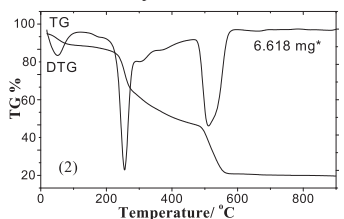

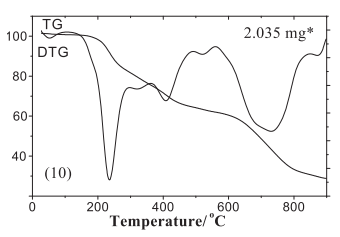


*: Weight of sample.

**Fig.S3. (TG/DTG) curves of the ligand (HL) and its metal complexes**

1. **Co (II), (2) Ni (II) and (10) Cd(II).**

**Table.S5.** The data for the thermal decomposition of the ligand (HL), and its Ni(II), Co(II) and Cd(II) complexes.

| **No** | **Compound** | TG range  (ºC) | DTG peak  (ºC) | Mass Loss % | | Assignment | TS  (ºC) |
| --- | --- | --- | --- | --- | --- | --- | --- |
|  |  |  |  | Found | Calcd. |  |  |
|  | **HL** | 25-133  133-505 | -  169^g^  201^c^  307^g^  611^I^  At 712 | -  55.47  42.20  0.57 | -  55.57  41.78  1.06 | stable  loss of C5H7N3SOd  loss of C7.75H11Nd  0.25C^r^ | 133 |
| **1** | **[Co(HL)Cl_2_H_2_O] 2.75 H_2_O** | 505-712  19-192  192-262  262-431  431-594 | 37^f^  216^c^  305^g^  529^c^  At 594 | 10.09  21.06  23.91  29.71  15.23 | 10.03  21.67  23.92  29.20  15.17 | loss of 2.75 mole of H_2_Oa  loss of Cl_2_ and 2 moles of H_2_O^d^  loss of C_8_H_8_N^d^  loss of C_5_H_10_N_3_S^d^  Co O^r^ | 192 |
| **2** | **[Ni(HL)Cl(H_2_O)_3_].Cl.3.5H_2_O** | 19-226  226-289  289-498  498-899 | 51^e^  255^c^  310^f^  512^c^  At 899 | 11.41  24.43  22.27  27.08  14.81 | 11.15  24.67  22.50  27.46  14.22 | loss of 3.25 mole of H_2_Oa  loss of Cl_2_ and 3.25 mole of H_2_O^d^  loss of C_8_H_8_N^d^  loss of C_5_H_10_N_3_S^d^  Ni O^r^ | 226 |
| **10** | **[Cd(HL)_2_Cl_2_].4EtOH.4H_2_O** | 29-166  166-607  607-899 | 51^g^  236^c^  408^f^  731^h^  At 899 | 1.45  49.21  38.52  10.82 | 1.16  49.17  38.39  11.28 | loss of 0.25 mole of EtOH^b^  loss of Cl_2_, 3.75 mole of EtOH, 4 mole of H_2_O and 2 moles of C_4_H_9_NO^d^  loss of C_18_H_18_N_6_S_2_^d^  Cd^r^ | 166 |

^a^: Dehydration, ^b^: Desolvation, ^d^: Decomposition, ^c^: strong, e: medium, ^f^: weak, g: very weak, ^h^: broad, ^I^: broad strong, ^r^: final residue.

All the results and discussion which had been presented in the supplementary file belonged to the published literature. [1]

**Reference**

1. El-Samanody, E.-S. A.; Polis, M. W.; Emara, E. M., Spectral studies, thermal investigation and biological activity of some metal complexes derived from (E)-N′-(1-(4-aminophenyl)ethylidene)morpholine-4-carbothiohydrazide. *Journal of Molecular Structure* **2017,** *1144*, 300-312.
